# Study of the Synergistic Immunomodulatory and Antifibrotic Effects of Dual-Loaded Budesonide and Serpine1 siRNA Lipid–Polymer Nanoparticles Targeting Macrophage Dysregulation in Tendinopathy

**DOI:** 10.1021/acsami.4c02363

**Published:** 2024-04-02

**Authors:** Sandra López-Cerdá, Giuseppina Molinaro, Rubén Pareja Tello, Alexandra Correia, Sarojinidevi Künig, Peter Steinberger, Michael Jeltsch, Jouni T. Hirvonen, Goncalo Barreto, Johannes Stöckl, Hélder A. Santos

**Affiliations:** † Drug Research Program, Division of Pharmaceutical Chemistry and Technology, University of Helsinki, Helsinki FI-00014, Finland; ‡ Centre for Pathophysiology, Infectiology and Immunology, Institute of Immunology, Medical University of Vienna, 1090 Vienna, Austria; § Individualized Drug Therapy Research Program, Faculty of Medicine, University of Helsinki, Helsinki FI-00014, Finland; ∥ Wihuri Research Institute, Helsinki FI-00014, Finland; ⊥ Helsinki One Health, University of Helsinki, Helsinki FI-00014, Finland; # Translational Immunology Research Program, Faculty of Medicine, University of Helsinki, Helsinki FI-00014, Finland; 7 Orton Orthopedic Hospital, Tenholantie 10, Helsinki 00280, Finland; 8 Medical Ultrasonics Laboratory (MEDUSA), Department of Neuroscience and Biomedical Engineering, Aalto University, Espoo 02150, Finland; 9 Department of Biomaterials and Biomedical Technology, University Medical Center Groningen, University of Groningen, Ant. Deusinglaan 1, 9713 AV Groningen, The Netherlands

**Keywords:** lipid−polymer hybrid nanoparticles, macrophages, tendinopathy, siRNA, dual drug delivery

## Abstract

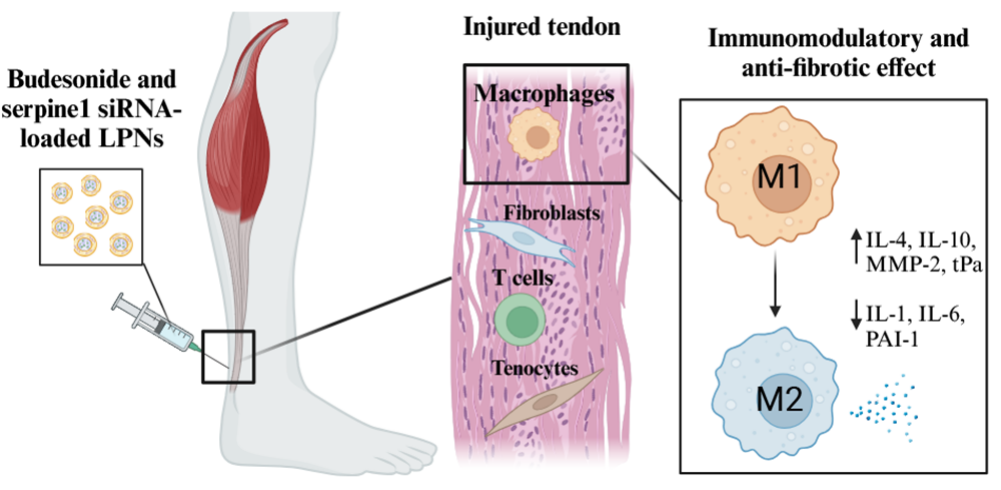

Musculoskeletal diseases involving tissue injury comprise tendon, ligament, and muscle injury. Recently, macrophages have been identified as key players in the tendon repair process, but no therapeutic strategy involving dual drug delivery and gene delivery to macrophages has been developed for targeting the two main dysregulated aspects of macrophages in tendinopathy, i.e., inflammation and fibrosis. Herein, the anti-inflammatory and antifibrotic effects of dual-loaded budesonide and serpine1 siRNA lipid–polymer hybrid nanoparticles (LPNs) are evaluated in murine and human macrophage cells. The modulation of the gene and protein expression of factors associated with inflammation and fibrosis in tendinopathy is demonstrated by real time polymerase chain reaction and Western blot. Macrophage polarization to the M2 phenotype and a decrease in the production of pro-inflammatory cytokines are confirmed in macrophage cell lines and primary cells. The increase in the activity of a matrix metalloproteinase involved in tissue remodelling is proven, and studies evaluating the interactions of LPNs with T cells proved that dual-loaded LPNs act specifically on macrophages and do not induce any collateral effects on T cells. Overall, these dual-loaded LPNs are a promising combinatorial therapeutic strategy with immunomodulatory and antifibrotic effects in dysregulated macrophages in the context of tendinopathy.

## Introduction

Musculoskeletal diseases (MSD) are among the main causes of impairment worldwide, causing pain and disabilities that affect daily activities and quality of life.^1^ Among MSD, tendinopathy is a complex tissue injury condition that affects sport practitioners and workers in specific occupational settings that involve forceful activities or repetitive movements.^2^ Specifically, tendinopathy accounts for 30–50% of MSD-related primary care visits worldwide, and the socioeconomic burden associated with tendinopathy is over EUR 180 billion in the United States and European Union, with a forecast of +25% increase over the next five years.^1,3,4^

Conventional therapies for tendon injury management are mainly based on physical therapy, the use of nonsteroidal anti-inflammatory drugs or ultrasound waves.^5−7^ Nevertheless, the limitations of these strategies are that they are not tissue-specific, they target only one of the aspects of tendinopathy, and they do not restore the original characteristics of the tissue.^8,9^ Moreover, while the effectiveness of certain drugs on other components of the musculoskeletal system has been proved, e.g., bisphosphonates in bone, myorelaxants in muscle, and anticonvulsants in peripheral nerve diseases, no specific tendon-target drugs have been developed.^10^ As a consequence, the systemic or oral administration of conventional anti-inflammatory drugs has been the most recurrent, but it is an unspecific approach that can lead to unsatisfactory delivery to the target tissue and to undesirable toxicities.^8,11^

Macrophages are immune cells that accumulate in the degenerating tendon and have been acknowledged as key regulators of the tendon healing process.^12,13^ In the first stages of tendon healing, macrophages acquire a pro-inflammatory M1 phenotype, leading to the secretion of some inflammatory cytokines, i.e., such as IL-1, IL-6, and to the upregulation of key inflammatory mediators such as NF-κβ1.^13,14^ In later stages of tendon repair, during the remodelling phase, macrophages shift to the pro-regenerative M2 phenotype and secrete anti-inflammatory molecules.^15^ Nevertheless, M2 macrophages upregulate TGF-β1, which leads to cell proliferation and excessive accumulation of extracellular matrix (ECM) components.^16,17^ As a consequence, collagen fibers align asymmetrically and scar tissue is formed, hampering the complete recovery of the tissue mechanical function.^18^ Therefore, macrophage function should be fine-tuned and modulated in order to promote the shift to the M2 phenotype to reduce inflammation but preventing the pro-fibrotic activity of M2 macrophages at the same time.^7^ Due to this, macrophages constitute a promising cell target for the design of novel therapeutics for tendinopathy, and the field of tendon regeneration could benefit from dual therapeutic approaches that target the most important aspects of macrophage dysregulation in tendon injury, i.e., inflammation and fibrosis.^19^

Nanoparticles (NPs) are in the forefront of the research concerning drug delivery.^20,21^ In the last years, the potential of NPs has been exploited by using safer and more efficient materials. For instance, biodegradable and biocompatible polymers have been employed to protect the drug payloads from degradation and allow the delivery of drugs in a more sustained and targeted manner.^22,23^ In addition, third-generation cationic lipids have been exploited to complex oligonucleotides and transfect cells for gene therapy purposes with low toxicity and immunogenicity.^20,24,25^ As a result of the combination of novel polymeric and lipidic materials, lipid–polymer hybrid nanoparticles (LPNs) have been proven to be an efficient nanoplatform for the coloading of drugs with different physicochemical properties.^26−29^ Recently, we demonstrated that newly optimized LPNs were successfully coloaded with an anti-inflammatory drug in the polymeric core and a model siRNA in the lipid shell using a newly developed microfluidics approach. These LPNs were not toxic, protected the drug cargoes from degradation, proved successful to control the release of the small molecule and the siRNA and efficiently transfected murine and human macrophage cell lines at low NP doses.^30^ Thus, the previously optimized LPNs constitute a suitable nanoplatform for the development of dual therapeutic approaches for the management of complex conditions, such as tendinopathy.

In this work, we propose the use of this platform of LPNs for developing a dual therapeutic approach targeting macrophages in tendinopathy. The optimized LPNs are loaded with a relevant anti-inflammatory small molecule drug, i.e., budesonide, and a relevant siRNA against the pro-fibrotic *Serpine1* gene, which encodes for plasminogen activator inhibitor 1 (PAI-1). On the one hand, budesonide is a corticosteroid that has been used for the treatment of inflammatory disease and has been successfully used for shifting macrophages from the M1 phenotype to the M2 phenotype in different conditions.^31−33^ The controlled delivery of budesonide by the developed LPNs is key because it allows for sustaining the release of this drug and enhancing the uptake by the target cells, thus avoiding repetitive administrations that could lead to long-term side effects.^33^ On the other hand, PAI-1 is a suppressor of fibrinolysis and protease activity that acts downstream of the TGF-β1 signaling pathway. Several works have described PAI-1 as a key pro-fibrotic factor involved in the formation of cell adhesion in tendinopathy, proposing PAI-1 as a more convenient therapeutic target in tendinopathy than TGF-β, since the effects of abolishing TGF-β are very wide and not all desirable.^18,33−35^ By abolishing PAI-1, the most deleterious effect of TGF-β1 upregulation, i.e., fibrotic tissue formation, can be avoided without affecting other beneficial effects of TGF-β1 upregulation, i.e., ECM formation and tenocyte growth.^18,35^ By harnessing the developed LPNs to deliver an siRNA against the *Serpine1* gene, the degradation of this sensitive molecule is prevented and the siRNA can be efficiently taken up by macrophages and escape the endosomal compartment to induce a potent gene silencing even at low LPNs concentrations, thus minimizing the immune activation associated with the delivery of nucleic acids.^30^

Upon loading the developed LPNs nanoplatform with the relevant payloads, the synergistic anti-inflammatory and antifibrotic effects of budesonide and serpine1 siRNA dual-loaded LPNs were tested in murine and human macrophage cell lines and in human primary macrophages. The modulation of the expression of genes and proteins related to inflammation and the TGF-β1/PAI-1 signaling pathway associated with fibrosis in tendon disease was assessed through different molecular biology techniques. In addition, immunological studies related with assessing the secretion of pro-inflammatory cytokines and macrophage polarization studies allowed us to examine the shift of macrophages to the M2 pro-regenerative phenotype in macrophage cell lines and primary cells. Moreover, a study assessing the activity of a matrix metalloproteinase (MMP) involved in ECM remodeling was used to demonstrate the potential of dual-loaded LPNs to enhance scarless tissue regeneration. Finally, further immunological studies were conducted to predict the lack of immunogenicity of the LPNs when interacting with T cells, which are also present in the immunological milieu of the regenerating tendon. The main goal of this study was to evaluate if dual-loaded LPNs could be used to resolve inflammation and prevent the expression of pro-fibrotic factors associated with tendinopathy on macrophages.

## Results and Discussion

### Physicochemical Characterization of BUD@siRNA@LPNs

The preparation of hybrid LPNs was previously optimized by a newly developed microfluidics method, and the safety of this nanoplatform, as well as the controlled release of the small molecule drug and a model siRNA, was proved *in vitro*.^30^ Here, LPNs were coloaded with the relevant anti-inflammatory drug budesonide (BUD), with the aim to shift M1 macrophages to the M2 pro-regenerative phenotype, and with serpine1 siRNA, to silence the expression of this tendon pro-fibrotic gene. The formulation and process parameters optimized to produce this nanoplatform were used for the preparation of the budesonide and serpine1 siRNA dual-loaded LPNs. As shown in Table 1, dual-loaded LPNs presented a size of 350 nm, which is suitable for local administration to the injured tendon, intramuscularly or subcutaneously. The polydispersity index (PDI) was 0.23, which confirms the particle homogeneity despite the dual drug loading, and the surface charge was +24 mV, which is due to the cationic lipid cKK-E12 in the lipid shell to complex the serpine1 siRNA. The encapsulation efficiency (EE) of the serpine1 siRNA was 68%, and the loading degree (LD) of BUD was 18% in the dual-loaded LPNs. This allows us to use the LPNs at the safe NP concentration of 100 μg/mL, which equals to 2 μg/mL BUD and 0.25 μg/mL siRNA, concentrations that have been previously reported to lead to therapeutic efficiency.^18,32^

**Table 1:** Characterization of the Size, PDI, Zeta Potential, Loading Degree (LD) and Encapsulation Efficiency (EE) of the Empty, Single-Loaded, and Dual-Loaded BUD and Serpine1 siRNA LPNs^a^

Formulation	Size (nm)	PDI	Zeta potential (mV)	Drug loading: EE/LD (%)
Empty LPNs	330 ± 5	0.2 ± 0.05	+24 ± 2	-
Serpine1 siRNA@LPNs	344 ± 4	0.2 ± 0.04	+24 ± 3	72% ± 10 EE
BUD@LPNs	350 ± 5	0.22 ± 0.03	+25 ± 2	20% ± 1.2 LD
Dual-loaded LPNs	347 ± 6	0.22 ± 0.05	+24 ± 3	18% ± 1.4 LD (BUD), 68% ± 11 EE (siRNA)

a Size, PDI, and zeta potential were analyzed by dynamic light scattering (DLS). The LD of budesonide was analyzed by a previously developed high performance liquid chromatography method (HPLC),^30^ and the EE of the serpine1 siRNA was analyzed using the Ribogreen assay. Data represent mean ± SD (*n* ≥ 3).

### Modulation of the Expression of Genes Related to Inflammation and Fibrosis in Tendinopathy by Dual-Loaded LPNs

The anti-inflammatory effect of budesonide and the antifibrotic effect of serpine1 siRNA coloaded in LPNs were evaluated at the gene level by real-time quantitative polymerase chain reaction (RT-qPCR). For this, the anti-inflammatory effect of BUD loaded in dual-loaded LPNs was assessed by analyzing the modulation of the gene expression of *Nfκb1*, *Tnfa* and *Tgfb1*. The antifibrotic effect of serpine1 siRNA loaded in dual-loaded LPNs was assessed by analyzing the expression of *Serpine1*, *tPa* and matrix metalloproteinase 2 (*Mmp2*). On the one hand, RAW 264.7 murine macrophage cells and human phorbol myristate acetate (PMA)-differentiated THP-1 cells were pretreated with LPS, and then BUD and BUD@LPNs were added for 24 and 48 h. On the other hand, cells were pretreated with murine or human TGF-β1 (which induces serpine1 overexpression), and then serpine1 siRNA and siRNA@LPNs were added for 24 and 48 h. These controls allowed us to assess the anti-inflammatory effect and the antifibrotic effect individually. For assessing the synergistic effect of the final formulation of dual-loaded LPNs, cells were pretreated with both LPS and TGF-β1 to induce an inflammatory and fibrotic profile in cells, and then dual-loaded LPNs were added for 24 and 48 h. Cells with no LPS or TGF-β1 pretreatment were treated with empty LPNs (100 μg/mL) for 24 and 48 h to assess the possible effects of the nanocarrier itself on the gene expression profile.

**Figure 1:**
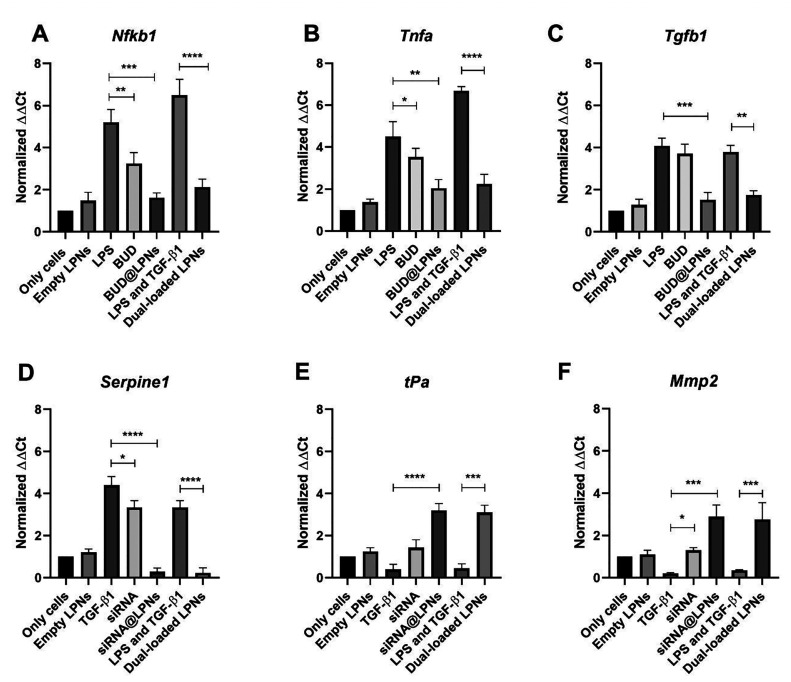
Evaluation of the expression of pro-inflammatory and pro-fibrotic genes by RT-qPCR in RAW 264.7 murine macrophage cells. The anti-inflammatory effect of budesonide and the antifibrotic effect of serpine1 siRNA have been evaluated in RAW 264.7 cells with BUD@LPNs, siRNA@LPNs, dual-loaded LPNs as well as the BUD and siRNA alone, by quantification of the gene expression of (A) *Nfkb1*, (B) *Tnfa*, (C) *Tgfb1*, (D) *Serpine1*, (E) *tPa* and (F) *Mmp2* after 24 h of treatment. Results are represented as fold increase values compared to the positive controls (LPS, TGF-β and LPS + TGF-β) ± SD (*n* ≥ 3). A one-way ANOVA followed by a Dunnett post-hoc test was used for the statistical analysis. The significance levels of the differences were set at the probabilities of *p < 0.05, **p < 0.01, ***p < 0.001, and ****p < 0.0001 for comparison with the positive control.

**Figure 2:**
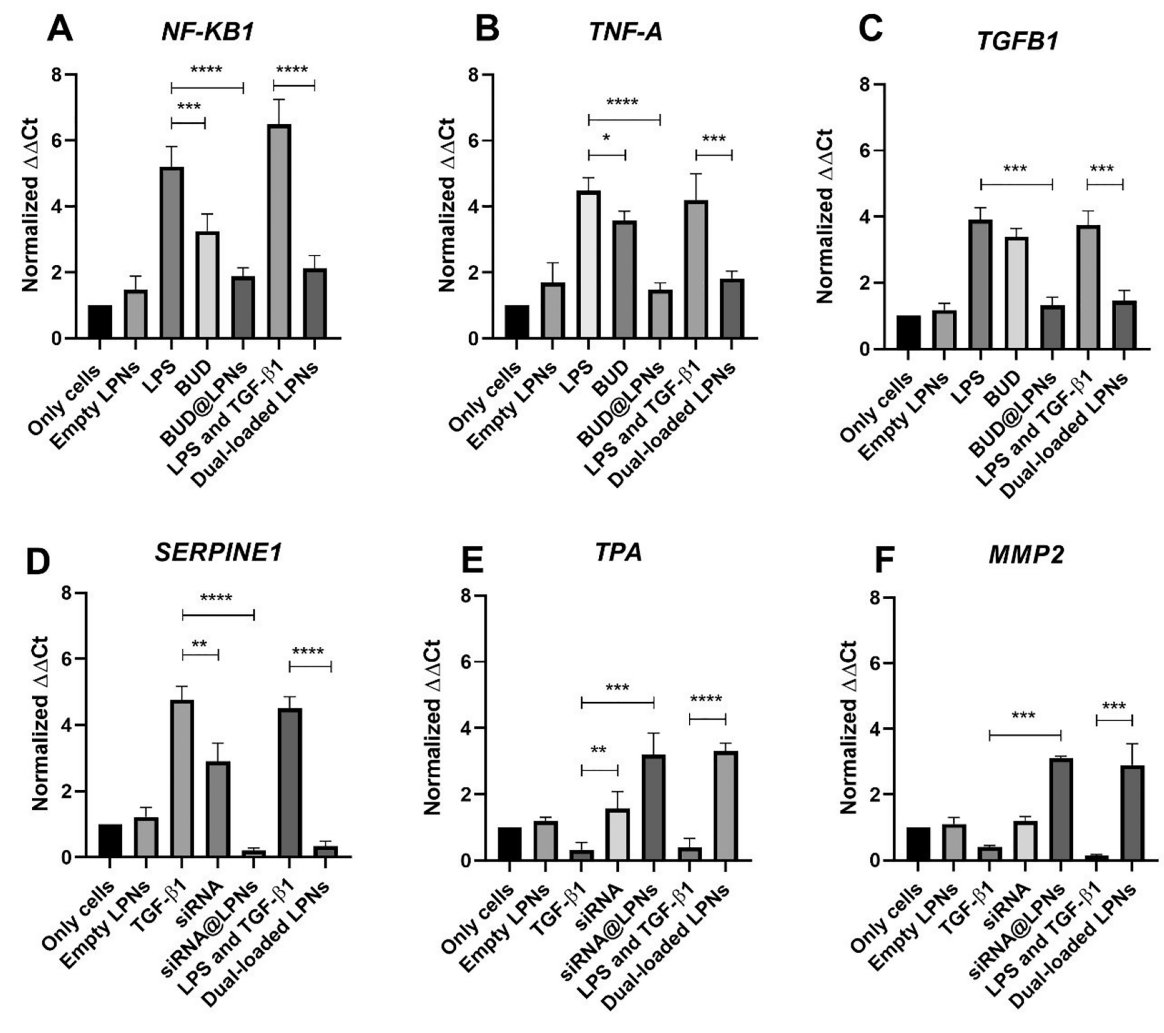
Evaluation of the expression of pro-inflammatory and pro-fibrotic genes by RT-qPCR in PMA-differentiated THP-1 cells. The anti-inflammatory effect of budesonide and the antifibrotic effect of serpine1 siRNA have been evaluated in THP-1 cells with BUD@LPNs, siRNA@LPNs, BUD@siRNA@LPNs as well as the BUD and siRNA alone, by quantification of the gene expression of (A) *NF-KB1*, (B) *TNFA*, (C) *TGFB1*, (D) *SERPINE1*, (E) *TPA* and (F) *MMP2* after 24 h of treatment. Results are represented as fold increase values compared to the positive controls (LPS, TGF-β and LPS + TGF-β) ± SD (*n* ≥ 3). A one-way ANOVA followed by a Dunnett post-hoc test was used for the statistical analysis. The significance levels of the differences were set at the probabilities of *p < 0.05, **p < 0.01, ***p < 0.001, and ****p < 0.0001 for comparison with the positive control.

On the one hand, NF-κβ and TNF-α are considered critical pathways in the regulation of pro-inflammatory cytokines’ production and apoptosis.^36^ In addition, TGF-β is another inflammatory mediator as well as a gene related with the formation of cell adhesions in later stages of the tendon healing process, since it is a gene upstream of the signaling pathway of PAI-1 (*Serpine1* gene).^35,37^ In Figure 1A–C and Figure 2A–C, the expression of *Nfkb1*, *Tnfa* and *Tgfb1* was downregulated significantly by both BUD@LPNs and dual-loaded LPNs as compared to the positive controls (LPS and LPS + TGF-β1, respectively) in both RAW 264.7 cells and THP-1 cells, respectively, after 24 and 48 h of treatment. The downregulation observed is also remarkably bigger than that observed with the anti-inflammatory compound (BUD) alone. This is explained by the fact that loading BUD in LPNs protects the drug from degradation, allowing a sustained release of the drug and improving the intracellular delivery, thus leading to an enhanced anti-inflammatory effect on macrophage cells.^32^

On the other hand, *Serpine1* is the gene encoding for PAI-1, an inhibitor of protease activity and fibrinolysis, which is involved in ECM accumulation and formation of fibrotic tissue and is the direct target of the siRNA loaded in LPNs.^38^ The expression of *Serpine1* is significantly downregulated when RAW 264.7 cells and PMA-differentiated THP-1 cells pretreated with TGF-β1 are treated with siRNA@LPNs and dual-loaded LPNs for 24 and 48 h, compared to the positive controls (TGF-β1 and LPS + TGF-β1, respectively) (Figures 1D and 2D). The strategy of silencing PAI-1 instead of the upstream mediator TGF-β1 is preferable since TGF-β1 has some beneficial effects on tendon healing, such as the induction of cell proliferation and ECM formation.^39^ Therefore, certain downregulation of TGF-β1 can be beneficial, but complete silencing is not desirable. Nevertheless, the silencing of its downstream mediator, PAI-1, allows for abolishing only the downside effect of TGF-β, which is the excessive formation of ECM in a disorganized manner.^40^ In addition, because of silencing *Serpine1*, the expression of *tPa* (downstream mediator of *serpine1*) was statistically significantly upregulated by both siRNA@LPNs and dual-loaded LPNs, meaning that the proteolytic activity of protease enzymes involved in tissue remodelling can be enhanced (Figures 1E and 2E).^18^ As a proof of that, in Figures 1F and 2F the expression of *Mmp2* is increased in both murine and human macrophages after 24 and 48 h of treatment, which confirms that silencing the key pro-fibrotic gene *Serpine1* can involve a potential increase in the activity of MMPs involved in ECM remodelling.^35^

### Modulation of the Protein Expression of Key Pro-inflammatory and Pro-fibrotic Mediators in Tendinopathy by Dual-Loaded LPNs

The changes in the gene expression do not always correlate with the changes in the protein expression since translation of an mRNA into a protein is a process independent and posterior to transcription.^41^ Therefore, the protein expression of NF-κβ1, the key pro-inflammatory mediator in tendinopathy, and the protein expression of TGF-β1, which plays both a pro-inflammatory and pro-fibrotic role in tendon healing, was evaluated by Western blot to demonstrate the anti-inflammatory and antifibrotic effect of BUD in the dual-loaded LPNs. In addition, intracellular staining was conducted to detect the changes in the protein production of PAI-1 intracellularly, and the activity of MMP-2, one of the MMPs regulated by PAI-1 and involved in ECM remodeling, was evaluated using a fluorescent-based assay after induction of fibrosis with TGF-β and treatment with the LPNs.

**Figure 3:**
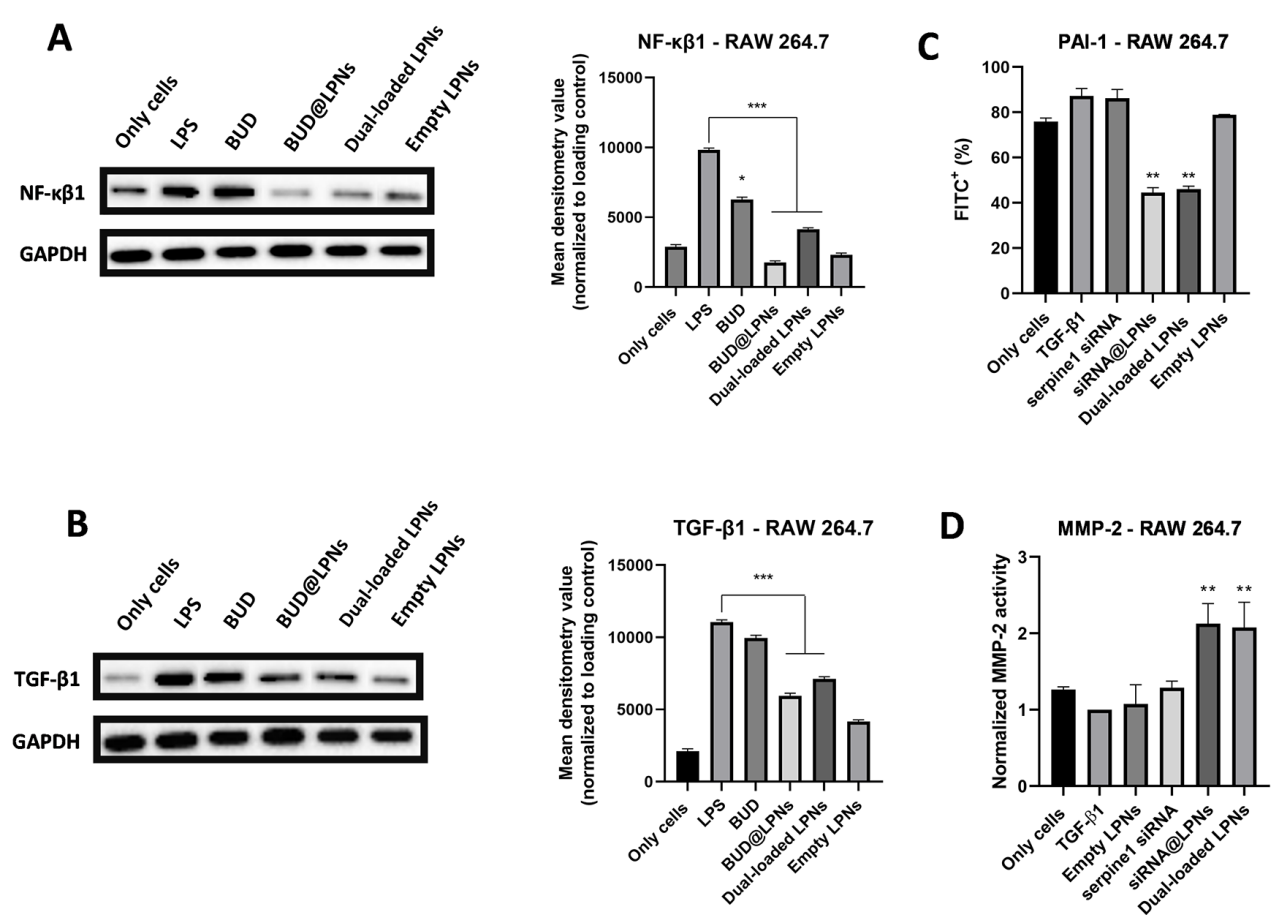
Protein expression analysis by Western blot of (A) murine NF-κβ1and (B) murine TGF-β1 in RAW 264.7 cells to study the anti-inflammatory effect of BUD in dual-loaded LPNs. Cells were pretreated with LPS (1 μg/mL) for 24 h and BUD, BUD@LPNs and dual-loaded LPNs were incubated for 48 h before cell lysis and protein extraction. Western blot bands are shown, and the mean densitometry value normalized to the loading control is represented as bar graphs on the right-hand side of the bands. (C) Assessment of the protein expression of PAI-1 (serpine1 gene) by intracellular staining after treating RAW 264.7 cells with siRNA@LPNs and dual-loaded LPNs. (D) Assessment of the enzymatic activity of MMP-2 by using a fluorescent MMP-2 substrate after treating RAW 264.7 cells with siRNA@LPNs and dual-loaded LPNs. In the bar graphs, a one-way ANOVA followed by a Dunnett post-hoc test was used for the statistical analysis. The significance levels of the differences were set at the probabilities of *p < 0.05, **p < 0.01 and ***p < 0.001 for comparison with the positive controls (LPS or TGF-β1).

As it can be seen in Figure 3A, the protein expression of NF-κβ1 was statistically significantly decreased after treating murine macrophages with single-loaded BUD@LPNs and dual-loaded LPNs. The effect of the single-loaded LPNs was more remarkable than that of the dual-loaded LPNs in murine macrophages but was not statistically significantly different. The effect of the drug alone was lower than that of the drug loaded in LPNs. Moreover, the empty LPNs did not show any further increase in the expression of NF-κβ1 in murine macrophages, which demonstrates that the nanocarrier itself does not trigger this inflammatory pathway. Similarly, we can see in Figure 3B that the expression of TGF-β1 in RAW 264.7 cells is decreased without being completely abolished. In fact, the aim of this therapeutic approach is not to completely abolish the production of TGF-β1, since this protein has pleiotropic effects during tendon healing, i.e., cell proliferation and matrix formation, that are beneficial for the tendon regeneration process.^17^

Next, the effects at the protein level of delivering serpine1 siRNA with LPNs were demonstrated by the statistically significant decrease in the protein expression of PAI-1 in RAW 264.7 cells pretreated with TGF-β1 (Figure 3C), which is in correlation with the gene expression data. In addition, the activity of MMP-2 after treating RAW 264.7 cells with dual-loaded LPNs was statistically significantly increased (Figure 3D), which confirms that the silencing of serpine1 has an effect on the expression of MMPs involved in matrix formation and remodelling, potentially aiding in fibrosis prevention, as previously reported.^16,18,42^

**Figure 4:**
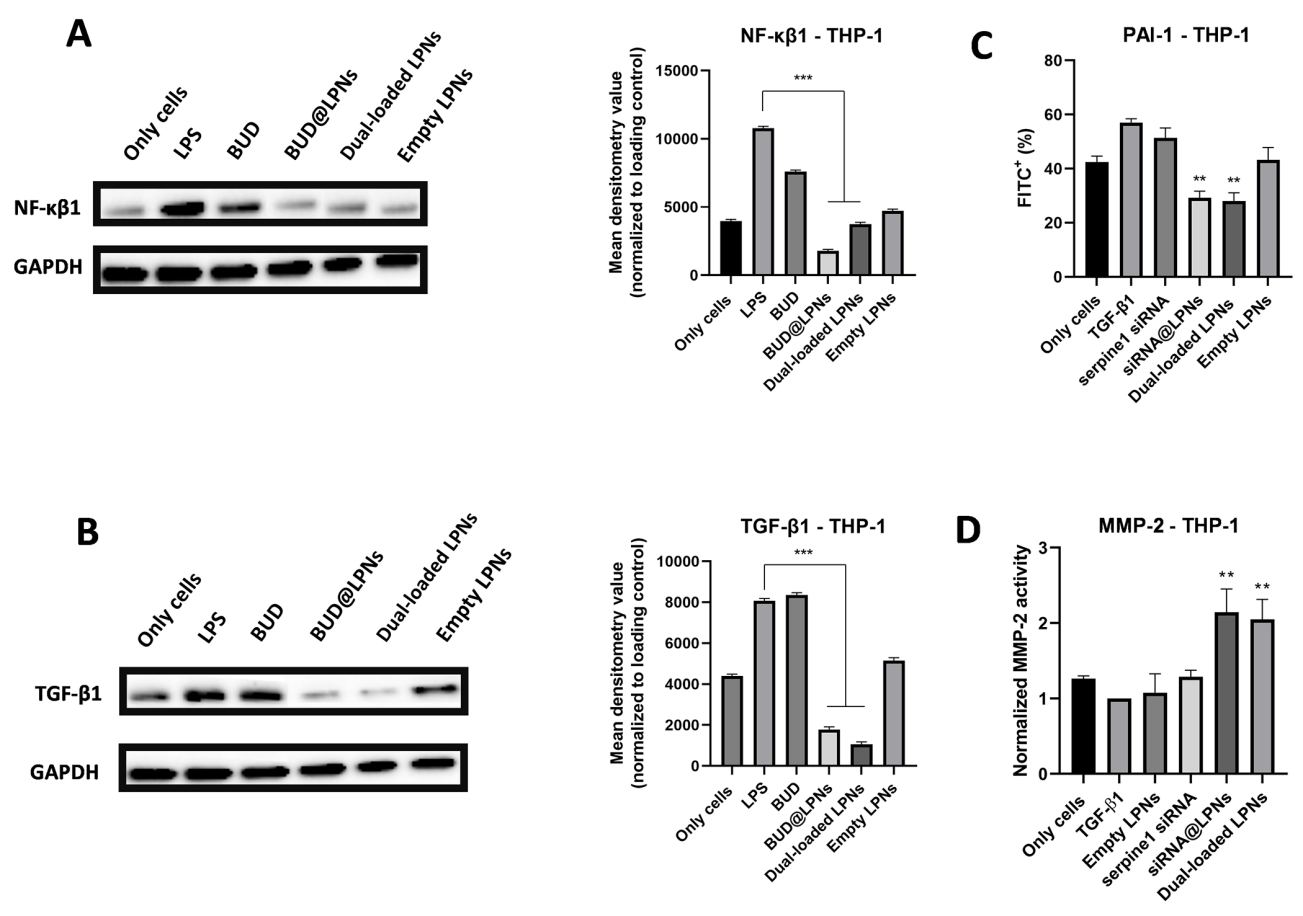
Protein expression analysis by Western blot of (A) human NF-κβ1 and (B) human TGF-β1 in PMA-differentiated THP-1 macrophage cells to study the anti-inflammatory effect of BUD in dual-loaded LPNs. Cells were pretreated with LPS (1 μg/mL) for 24 h and BUD, BUD@LPNs and dual-loaded LPNs were incubated for 48 h before cell lysis and protein extraction. Western blot bands are shown, and the mean densitometry value normalized to the loading control is represented as bar graphs on the right-hand side of the bands. (C) Assessment of the protein expression of PAI-1 (serpine1 gene) by intracellular staining after treating THP-1 cells with siRNA@LPNs and dual-loaded LPNs. (D) Assessment of the enzymatic activity of MMP-2 by using a fluorescent MMP-2 substrate after treating THP-1 cells with siRNA@LPNs and dual-loaded LPNs. In the bar graphs, a one-way ANOVA followed by a Dunnett post-hoc test was used for the statistical analysis. The significance levels of the differences were set at the probabilities of *p < 0.05, **p < 0.01 and ***p < 0.001 for comparison with the positive controls (LPS or TGF-β1).

Generally, similar results were obtained in PMA-differentiated THP-1 macrophage cells. As shown in Figure 4A, the NF-κβ1 downregulation by single-loaded BUD@LPNs and dual-loaded LNPs can also be proven in human THP-1 cells, but a more remarkable decrease in TGF-β1 protein expression is observed after treatment with the BUD@LPNs and dual-loaded LPNs in the case of PMA-differentiated THP-1 macrophage cells compared to RAW 264.7 (Figures 4B and 3B). In addition, when tested in THP-1 cells, both single-loaded siRNA@LPNs and dual-loaded LPNs also decreased the protein expression levels of PAI-1 (Figure 4C), even if the levels of expression of this protein were overall lower in this cell line. Furthermore, MMP-2 activity was also enhanced in THP-1 cells to a similar extent as it was observed in RAW 264.7 cells (Figure 4D). In conclusion, the modulation of tendinopathy-relevant pro-inflammatory and pro-fibrotic genes was also demonstrated at the protein level in both murine RAW 264.7 cells and in human PMA-differentiated THP-1 cells.

### Shift of Macrophages to the M2 Pro-regenerative Phenotype and Modulation of the Production of Cytokines in Macrophage Cell Lines

The M2 macrophages phenotype has been associated with resolution of inflammation and tendon tissue healing through increased tissue deposition.^7^ In addition, M2 macrophages release several anti-inflammatory mediators like IL-1 receptor antagonist and IL-4 as well as growth factors such as TGF-β. BUD is a corticosteroid that has been described to switch macrophages to the M2 phenotype in other therapeutic applications.^7^ Even if budesonide has been ascribed some long-term side effects, the delivery of this drug with LPNs allows us to reduce the dose needed for therapeutic efficacy and to avoid repeated administrations.^32,33,43^ Hence, the potential of BUD loaded in LPNs to shift macrophages from an M1 inflammatory profile to the pro-regenerative M2 phenotype was evaluated by analyzing the expression of the M1 marker CD86 and the M2 marker CD206 by antibody staining and flow cytometry analysis following the gating strategy in Scheme S1. In addition, changes in the release of the pro-inflammatory cytokine IL-1β and changes in the release of the anti-inflammatory cytokine IL-4 were studied by enzyme-linked immunosorbent assay (ELISA) in the supernatants collected.

**Figure 5:**
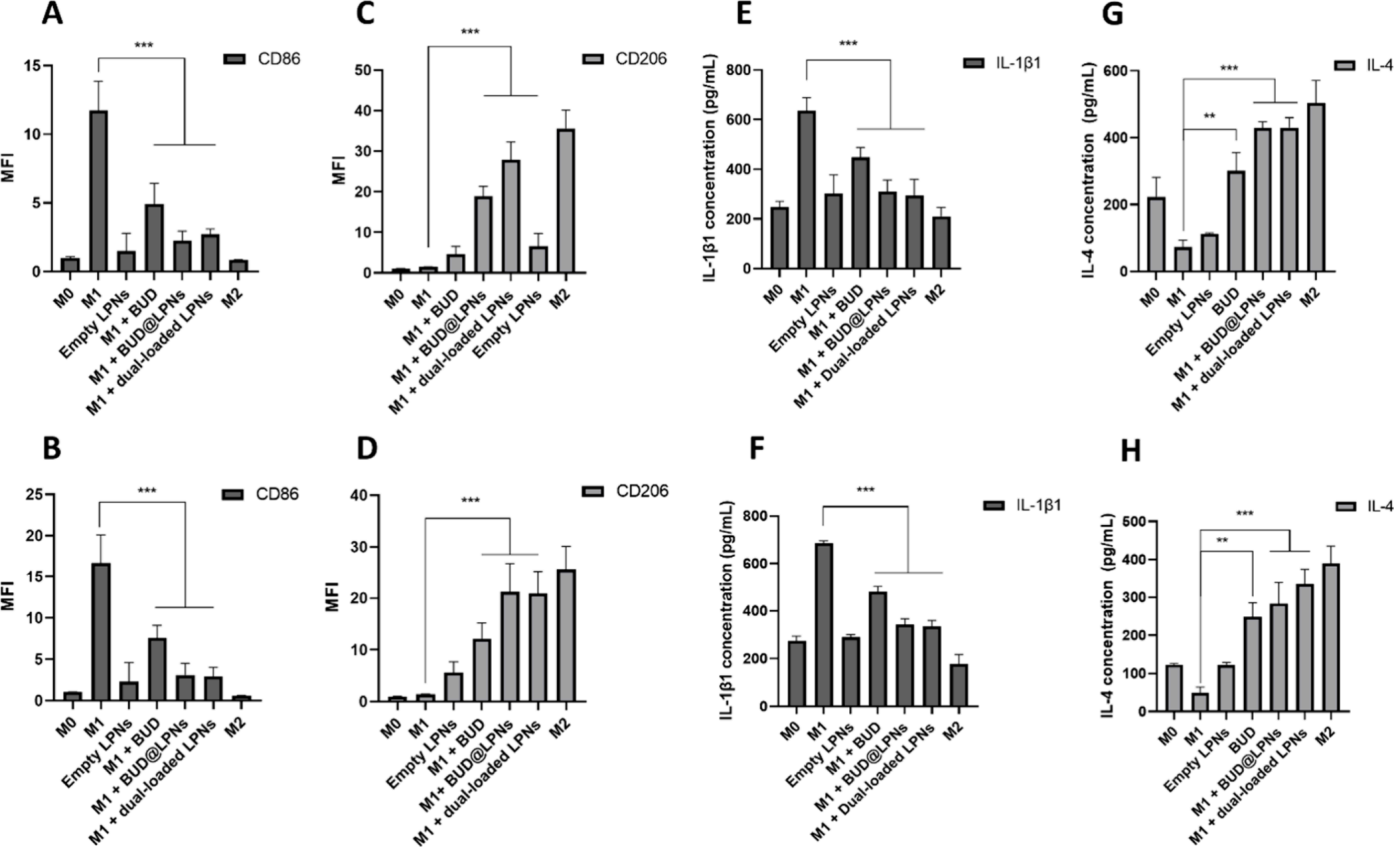
Macrophage polarization study with BUD and serpine1 siRNA dual-loaded LPNs in murine and human macrophage cell lines. Flow cytometry analysis of macrophage markers CD86 and CD206 expression after immunostaining of (A, C) RAW 264.7 cells and (B, D) PMA-differentiated THP-1 cells. The MFI was plotted compared with nonstained samples. Concentrations of IL-1β1 and IL-4 in the macrophage culture medium of (E, G) RAW 264.7 cells and (F, H) THP-1 cells after stimulation and treatment were quantified by ELISA. Data are presented as the mean ± SD (*n* = 3). Pro-inflammatory factors are shown in dark gray, and anti-inflammatory factors are shown in light gray. (A, C, E, G) Data for RAW 264.7 cells, and (B, D, F, H) data for THP-1 cells. A one-way ANOVA followed by a Dunnett post-hoc test was used for the statistical analysis. The significance levels of the differences were set at the probabilities of **p < 0.01 for comparing the treatment samples with the M1 positive control, *p < 0.05, **p < 0.01 and ***p < 0.001.

The treatment of M1 macrophages with dual-loaded LPNs led to a decrease in the expression of the CD86 marker in both murine RAW 264.7 cells and PMA-differentiated THP-1 cells (Figure 5A and B), while the expression of the M2 marker CD206 was significantly enhanced upon treatment with dual-loaded LPNs (Figure 5C and D) in both cell lines. BUD alone was also able to decrease the expression of CD86 and enhance the expression of CD206, but not as remarkably as in the case of the drug loaded in the LPNs (Figure 5A–D). M1 macrophages treated with dual-loaded LPNs produced lower amounts of IL-1 than the M1 control (Figure 5E and F), higher amounts of IL-4 than the M1 control, and similar levels of IL-4 than the M2 control in both cell lines tested (Figure 5G and H). In addition, no statistically significant differences were observed between the single-loaded BUD@LPNs and the dual-loaded LPNs, which proves that coloading siRNA into the lipid shell of the LPNs does not affect the release and therapeutic efficacy of BUD. These results confirmed that loading BUD into LPNs leads to a superior effect than the drug alone at the lower dose of 2 μg/mL, when it comes to promoting the shift of macrophages to the M2 pro-regenerative phenotype.

### Shift of Macrophages to the M2 Pro-regenerative Phenotype and Modulation of the Production of Cytokines in Human Primary Macrophages

**Figure 6:**
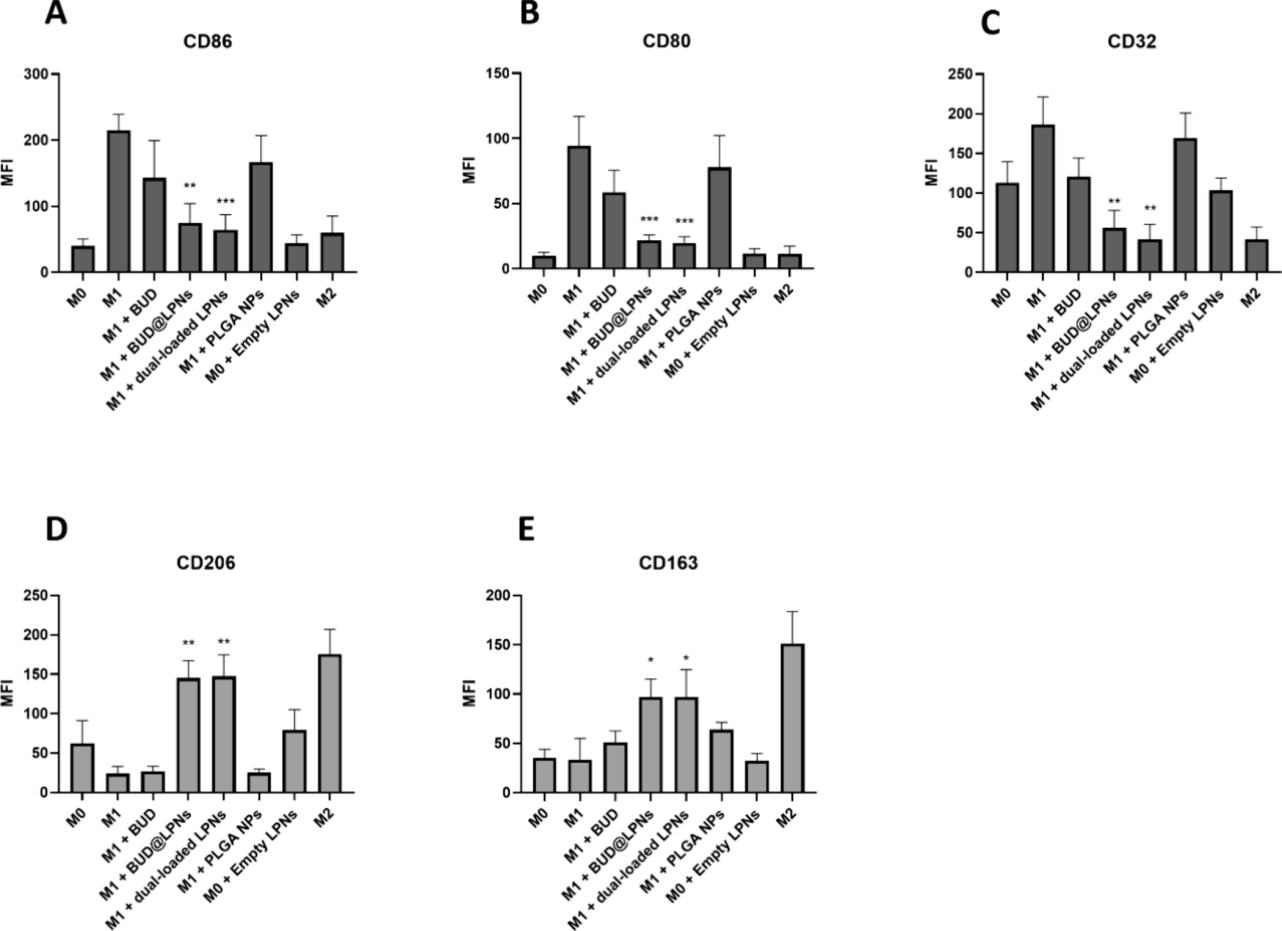
Macrophage polarization study with BUD and serpine1 siRNA dual-loaded LPNs in human primary macrophages. Flow cytometry analysis of macrophage M1 markers (A) CD86, (B) CD80 and (C) CD32 (in dark gray) and analysis of the expression of macrophage M2 markers (D) CD206 and (E) CD163 (in light gray) after immunostaining of human primary macrophages. The MFI was plotted compared with nonstained samples. Data are presented as the mean ± SD (*n* = 3 biological replicates). A one-way ANOVA followed by a Dunnett post-hoc test was used for the statistical analysis. The significance levels of the differences were set at the probabilities of **p < 0.01 for comparing the treatment samples with the M1 positive control, *p < 0.05, **p < 0.01 and ***p < 0.001.

Previous works have shown that different outputs can be obtained from macrophage polarization studies conducted in cell lines compared to primary cells.^32,44^ For this, it is essential to confirm the macrophage polarization data obtained in RAW 264.7 cells and PMA-differentiated THP-1 cells by testing the LPNs in primary human macrophages. In this case, all the well-established M1 and M2 characteristic surface markers were studied upon treating human primary macrophages with LPS and IFN-**γ**, and then with the single-loaded LPNs, dual-loaded LPNs and BUD alone.^45^ The expression of the M1 markers CD86, CD80 and CD32 and of the M2 markers CD206 and CD163 was analyzed by flow cytometry after staining with the corresponding antibodies, using the gating strategy in Scheme S1. In Figures 6 and S1, the CD80, CD86 and CD32 were upregulated in the M1 controls, while the expression in M2 was remarkably lower. In addition, the M2 control displayed much higher expression of the classical M2 markers CD206 and CD163 as compared to the M1 control, proving that the differentiation protocol worked.^45^ When the dual-loaded LPNs were added to macrophages treated with LPS and IFN- **γ**, the expression of the M1 markers CD80, CD86 and CD32 was statistically significantly decreased and the expression of the M2 markers CD206 and CD163 was statistically significantly increased compared to the M1 control (Figures 6 and S1). This occurred similarly with single-loaded BUD@LPNs, meaning that the coloading of the two drugs does not prevent the release and effect of BUD (Figure 6). Furthermore, the release of the pro-inflammatory cytokines IL-1β1, IL-6 and IL-12 was quantified in the supernatants of the samples collected for flow cytometry analysis. As we can see in Figure S2, the concentration of these cytokines was highly decreased to levels similar to those in the M2 control samples in the supernatants of M1 macrophages treated with the single-loaded BUD@LPNs and dual-loaded LPNs, further supporting the shift of human primary macrophages to the anti-inflammatory phenotype.

In addition, a coculture assay using human primary macrophages and allogenic T cells was set up to indirectly evaluate the phenotype of the human primary macrophages pretreated with LPNs. When allogenic T cells are cocultured with human primary macrophages, T cells start to proliferate due to the antigen presenting function of macrophages. Previously, it was observed that the proliferation of T cells follows a different profile when establishing a coculture of M1 macrophages with allogenic T cells vs a coculture of M2 macrophages and T cells.^46^ When the number of M2 macrophages in coculture with T cells is increased, the proliferation of T cells increases to a higher extent than that of T cells in coculture with M1 macrophages.^46^ Based on this, a fixed number of T cells was put in coculture with different numbers of macrophages, and the proliferation rate profile of T cells in coculture with M1 macrophages treated with dual-loaded LPNs was compared to that of T cells in coculture with M0, M1 and M2 macrophages (controls).

**Figure 7:**
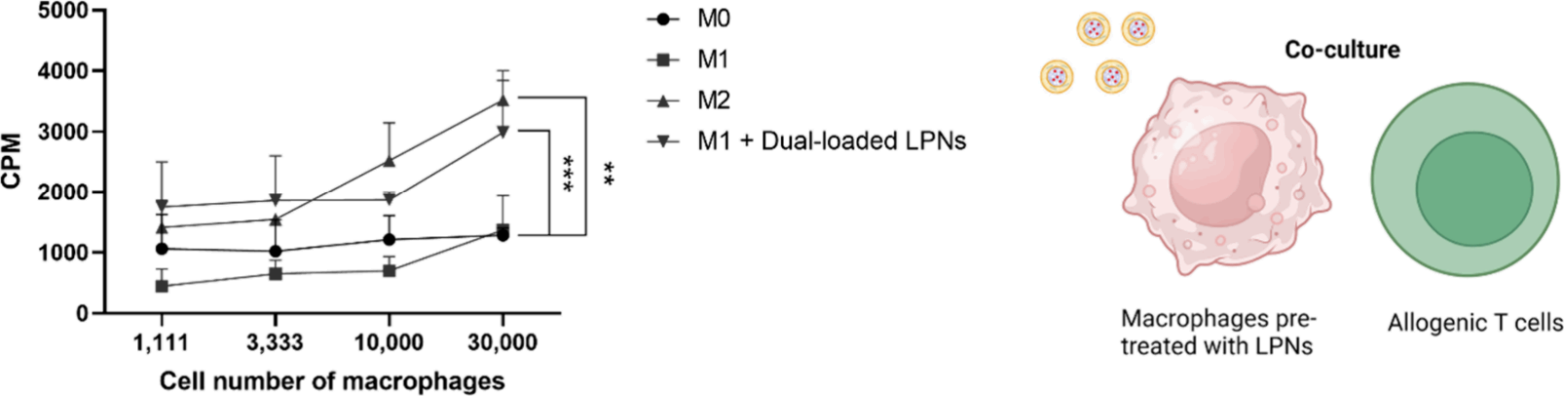
Schematic of the coculture of allogenic T cells and macrophages pretreated with LPNs and proliferation rate of allogenic T cells in coculture with human primary macrophages pretreated with dual-loaded LPNs. Different numbers of macrophages pretreated with dual-loaded LPNs were put in coculture with a fixed number of allogenic T cells to assess the proliferation profile of T cells in coculture with M1 + dual-loaded LPNs vs the M0, M1 and M2 controls. A radioactivity-based assay was used to measure the proliferation rate of T cells. The counts per minute (CPM) were measured using a Beta counter. Data is represented as mean ± SD of *n* = 3 biological replicates. A one-way ANOVA followed by a Dunnett post-hoc test was used for the statistical analysis. The significance levels of the differences were set at the probabilities of **p < 0.01 for comparing the M0, M2 and M1 + dual-loaded LPNs samples with the M1 positive control, *p < 0.05, **p < 0.01 and ***p < 0.001.

As shown in Figure 7, increasing the number of M0 and M1 macrophages did not statistically significantly affect the proliferation rate of T cells, represented as counts per minute (CPM). In addition, the proliferation rate in these cases is lower than that of T cells in coculture with M2 and M1 + dual-loaded LPNs at all macrophage numbers tested. Furthermore, the tendency of an increasing proliferation rate of T cells as the number of M2 macrophages is increased was also observed with M1 macrophages treated with dual-loaded LPNs. This observation confirms that the M1 macrophages treated with the dual-loaded LPNs have similar behavior to M2 cells, further confirming the efficiency of dual-loaded LPNs in shifting the macrophage phenotype and also confirming that this coculture study is a suitable test to evaluate the potential of nanosystems to shift the macrophage phenotype.

### Evaluation of the Interactions of Dual-Loaded LPNs with Reporter T Cells

One of the purposes of these dual-loaded LPNs is to fine-tune the response of macrophages present in the regenerating tendon tissue by inducing immunomodulation and promoting the resolution of inflammation. The immunological milieu present in the tendon tissue is composed of macrophages but also other immune cells like T cells.^47^ T cells can get activated through different pathways involving toll-like receptors (TLRs) signaling when in contact with agonists, leading to the activation of factors like NF-κβ1.^48,49^ For example, TLR4 can recognize lipopolysaccharides, TLR2/1, TLR2/6 and TLR2/1/6 can recognize lipoproteins and lipopeptides, and TLR7/8 can recognize RNA molecules.^49−51^ In our therapeutic context, the potential activation of TLRs is not desirable and therefore needs to be evaluated since the developed LPNs are constituted by lipids and are loaded with an siRNA.

For this purpose, reporter Jurkat T cells transformed with plasmids to express exclusively TLR4, TLR2/1, TLR2/6 and TLR 2/1/6 individually, were used to assess if some of the LPNs components or the payloads activate TLR signaling.^52^ The reporter T cells used are transformed with a plasmid that allows the expression of enhanced green fluorescence protein (eGFP) only if NF-κβ1 is activated upon TLR signaling (Scheme S2).^52^ In addition, reporter THP-1 monocytes that express TLR1/2, TLR2/4, TLR2/6, TLR3 and TLR7/8 and express eGFP upon NF-κβ1 activation were used as a model to perform a wider assessment of the induction of TLR signaling by the LPNs and the loaded drugs.^53^ The different reporter T cell lines and THP-1 cells were put in contact with dual-loaded LPNs, single-loaded LPNs and the drugs for 24 and 48 h, when the eGFP expression was measured.

**Figure 8:**
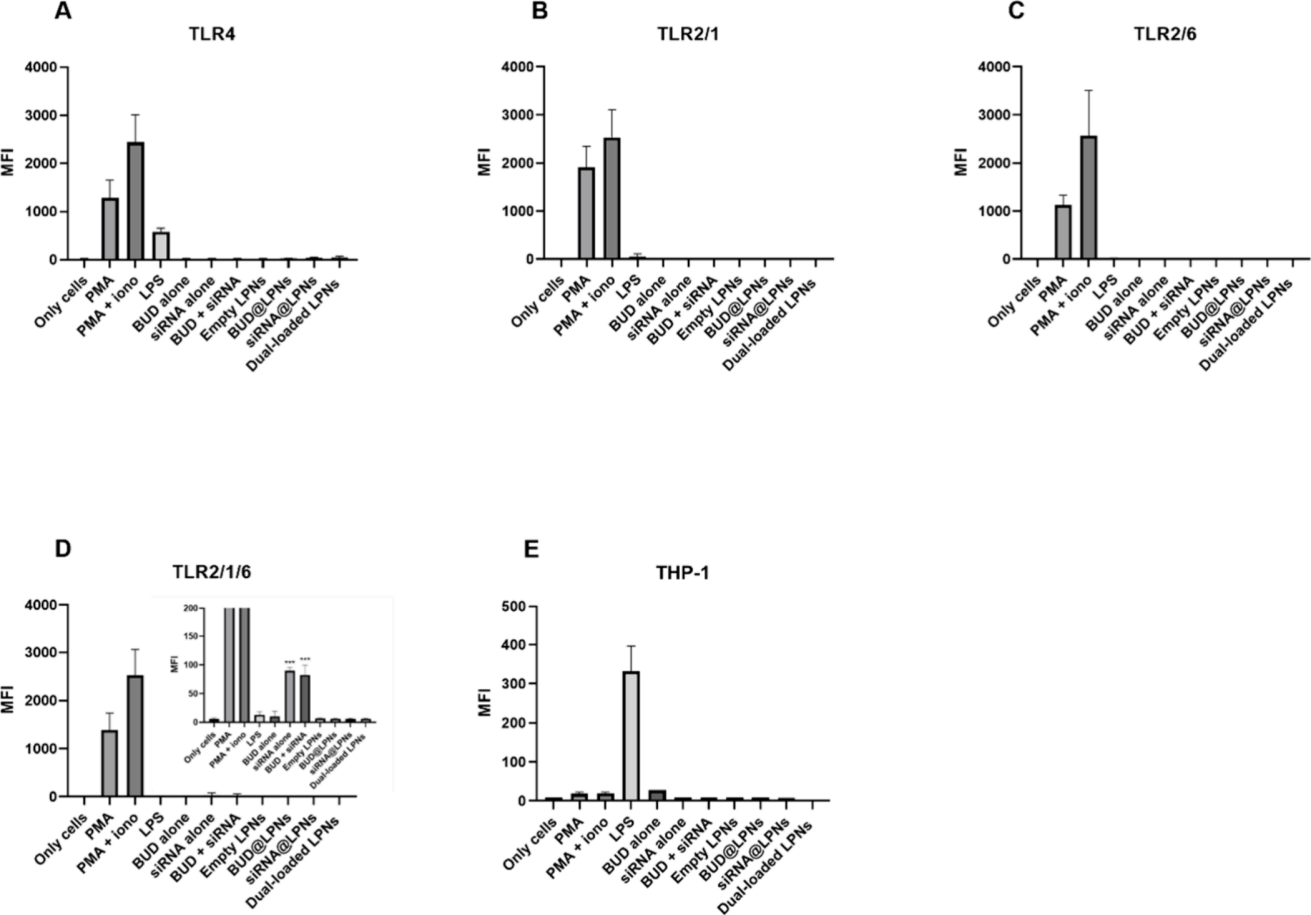
Activation of toll-like receptors (TLRs) in reporter T cells and reporter THP-1 monocytes by single-loaded LPNs, dual-loaded LPNs and the drugs (BUD and serpine1 siRNA alone). The indicated cells were incubated for 24 h with the corresponding LPNs/drugs, and cells were harvested to analyze the expression of eGFP as an indicator of TLR signaling activation. Phorbol myristate acetate (PMA) and PMA + ionomycin were used as positive controls for (A) TLR4, (B) TLR2/1, (C) TLR2/6 and (D) TLR2/1/6 reporter T cell lines, and LPS was used as a positive control for (E) reporter THP-1 monocytes. Data represent the mean fluorescence intensity (MFI) ± SD (*n* = 3). A one-way ANOVA followed by a Dunnett post-hoc test was used for the statistical analysis. The significance levels of the differences were set at the probabilities of *p < 0.05, **p < 0.01 and ***p < 0.001, to compare the negative control (only cells) with the treatment samples.

In Figure 8, it is shown that the reporter T cell system works properly since high levels of eGFP expression are observed when PMA, PMA + ionomycin, and/or LPS are used, meaning that these are suitable positive controls for comparison with LPNs and its components. However, when the cells are incubated with LPNs for 24 h, no expression of eGFP is measured in any of the TLR reporter cells (Figure 8A–E). In the case of the siRNA alone, certain activation of TLR2/1/6 was observed when the siRNA alone and siRNA + BUD alone are compared to the negative control (only cells), but this activation is much less remarkable than the activation induced by the positive controls (Figure 8D). The reason why this formulation of dual-loaded LPNs is not activating the TLRs under study is most probably the formulation design. For example, Foged et al. have confirmed that the formulation of cationic lipidoids (e.g., cKK-E12 used in these LPNs) led to the activation of TLR4, but when these lipidoids are formulated into lipid–PLGA hybrid NPs, the activation of TLR4 is abrogated.^49^ Similar results were obtained when the reporter T cells were incubated with the LPNs for 48 h (Figure S3). However, at this time point, the siRNA alone and in combination with BUD alone were activating THP-1 cells significantly (Figure S3E), while the siRNA@LPNs and dual-loaded LPNs did not, highlighting the importance of encapsulating the siRNA in LPNs to prevent the activation of TLRs like the TLR7/8 expressed by these cells.^51,53^ Overall, it was shown that this formulation of LPN and its components do not activate TLRs signaling in the indicated reporter cells, and the encapsulation of siRNA into these LPNs avoids the activation of TLRs by the siRNA. Hence, the importance of the formulation design on the biological effects of nanoplatforms is highlighted. Morever, it was demonstrated that this assay can be included as a standard test in the pipeline for the development of novel nanoplatforms used with immunomodulatory purposes.

Since LPNs did not activate TLR signaling in T cells, we aimed to further test if LPNs can inhibit already activated T cells, which could be an additional immunomodulatory effect of these LPNs acting not only on macrophages but on T cells. For this purpose, an assay based on the use of triple reporter Jurkat T cells transformed with plasmids that report the expression of eGFP, CFP and mCherry upon NFAT, NF-κβ and AP.1 activation, respectively, was set up.^54^ The three transcription factors reported by these cells regulate the expression of genes involved in the immune activation in response to a variety of stimuli, including cytokines, growth factors, stress, and bacterial and viral infections.^54^ Therefore, these T cells were activated by precoating the culture plates with CD3 and CD3 + CD28 antibodies, and the preactivated T cells were treated with LPNs and its components to evaluate any possible inhibitory effect of the developed LPNs.

**Figure 9:**
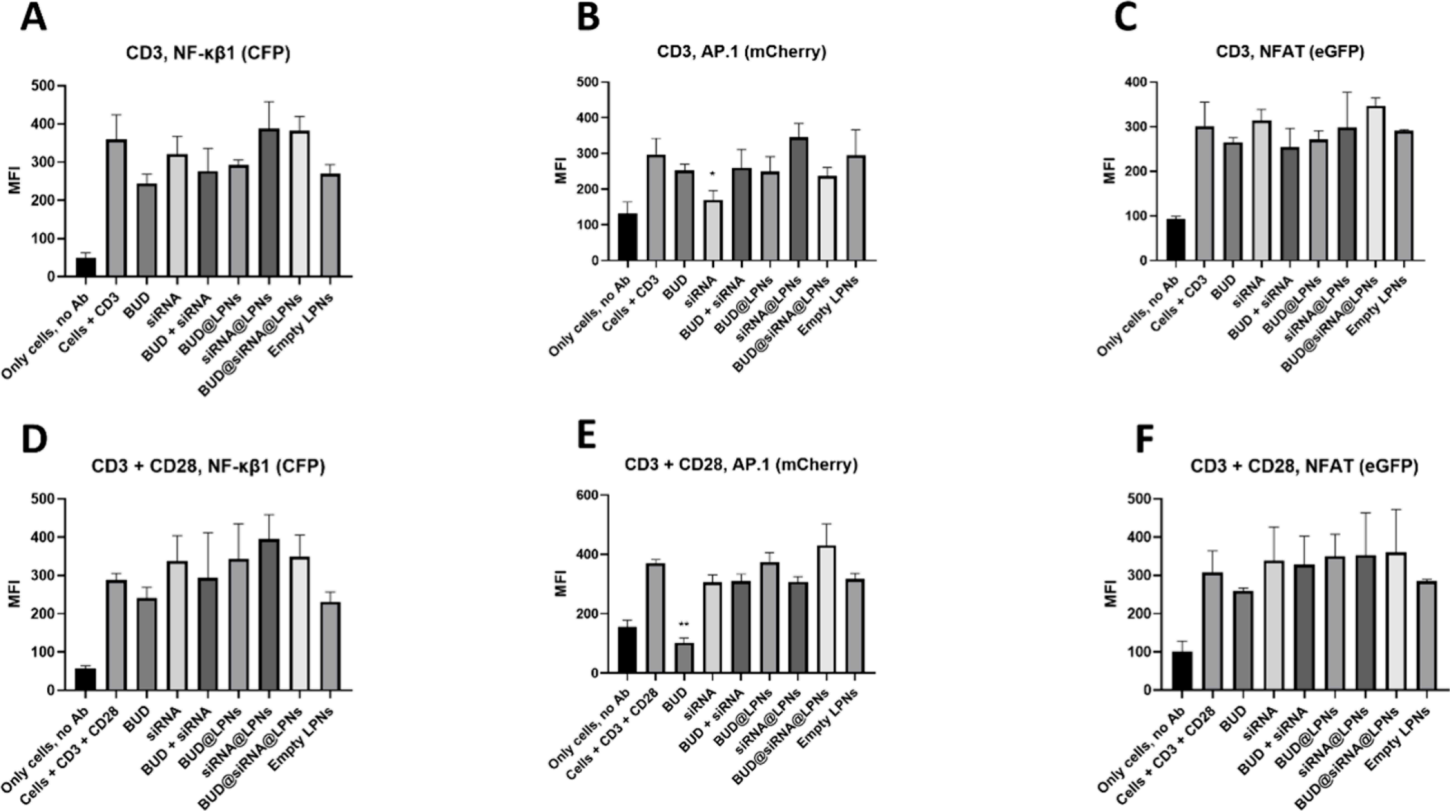
Assessment of the activation status of preactivated triple reporter Jurkat T cells after incubation with the single-loaded LPNs, dual-loaded LPNs and BUD and serpine1 siRNA alone for 48 h. The activation of the transcription factors NF-κβ1, AP.1 and NFAT was evaluated in CD3-preactivated T cells (A, B, C) and CD3 + CD28-preactivated T cells (D, E, F) after incubating the cells with the LPNs or the drugs for 48 h. Data represent the mean fluorescence intensity (MFI) ± SD (*n* = 3). A one-way ANOVA followed by a Dunnett post-hoc test was used for the statistical analysis. The significance levels of the differences were set at the probabilities of **p < 0.01 for comparing activated T cells samples (cells + CD3 and cells + CD3 + CD28) with the samples of LPNs and drugs, *p < 0.05, **p < 0.01 and ***p < 0.001.

In Figure 9, the intensity of the expression of CFP, mCherry, and eGFP from the different transcription factors is expressed as fluorescence intensity in MFI units measured by flow cytometry. The results show that the values of MFI of the samples treated with LPNs (empty and loaded) and the drugs alone are comparable to those of the positive controls (cells activated with CD3 antibody or CD3 + CD28 antibodies), and that only the siRNA alone and BUD alone decreased to some extent the activation of the AP.1 transcription factor in CD3 preactivated T cells and CD3 + CD28 preactivated T cells, respectively (Figure 9B and E). However, the dual-loaded LPNs did not decrease the activation of any of the transcription factors under study. With the data of this and the previous study with reporter cells, we can confirm that dual-loaded LPN and its components neither activate T cells nor inhibit already activated T cells, indicating that their immunomodulatory effects are directed toward macrophages and do not affect T cells.

## Conclusions

Here, an LPNs platform designed for the coloading of drugs is loaded with budesonide and serpine1 siRNA to test it as a dual therapeutic approach to target the two main dysregulated aspects of macrophages in tendinopathy, i.e., inflammation and fibrosis. The anti-inflammatory, immunomodulatory, and antifibrotic effects of LPNs are studied in depth in murine and human macrophages using molecular biology techniques. We demonstrated that key pro-inflammatory genes and proteins are downregulated by the treatment with the dual-loaded LPNs and M1 macrophages can be shifted to the M2 phenotype. Furthermore, the pro-fibrotic tendon gene serpine1 and its corresponding PAI-1 protein are downregulated, leading to enhanced expression of ECM remodelling factors. In addition, LPNs demonstrated to not have any collateral immunological effects, proving its suitability to be used with immunomodulatory purposes. Overall, budesonide and serpine1 siRNA dual-loaded LPNs could constitute a potential therapeutic option in the early stages of tendon disease in order to resolve inflammation and promote scarless tendon repair.

## Materials and Methods

### Materials for LPNs Preparation

PLGA PURASORB PDLG 5004A (50/50 d,l-lactide/glycolide copolymer) was kindly gifted by Corbion. Fluorescein isothiocyanate (FITC)-labeled PLGA was obtained from Nanosoft Polymers (NC, U.S.A.). cKK-E12 was purchased from Echelon Bioscience (Salt Lake City, Utah). 1,2-Distearoyl-*sn*-glycero-3-phosphocholine (DSPC) and cholesterol were obtained from Avanti Polar Lipids (Alabaster, AL, U.S.A.). Human and murine serpine1 siRNA were obtained from Eurogentec (Seraing, Belgium), and budesonide (BUD) was purchased from TCI (Tokyo, Japan). Diethyl pyrocarbonate (DEPC) and poly(vinyl alcohol) (PVA) (MW, 31,000–50,000 g mol^–1^) were purchased from Sigma-Aldrich (St. Louis, MO, U.S.A.). Quant-iT RiboGreen RNA Reagent and Tris–EDTA buffer (10 mM Tris, 1 mM EDTA, pH 8.0) (TE Buffer) were obtained from Molecular Probes, Invitrogen (Paisley, U.K.).

### Materials for Cell Biology Studies

RAW 264.7 and THP-1 macrophage cells were obtained from the American Type Culture Collection (ATCC, U.S.A.). Jurkat JE6-1 TLR 4, TLR 6, TLR 2/1, TLR 2/6 and TLR 2/1/6 were a kind gift from Peter Steinberg’s Lab (Medical University of Vienna). Hank’s balanced salt solution (HBSS), Dulbecco’s modified Eagle’s medium (DMEM), Roswell Park Memorial Institute (RPMI) 1640, fetal bovine serum (FBS), phorbol 12-myristate 13-acetate (PMA) and ionomycin (iono) was purchased from Sigma-Aldrich. Trypsin-ethylenediaminetetraacetic acid (EDTA) was purchased from Invitrogen, U.S.A.

### RT-qPCR

Empty, single-loaded, and budesonide and serpine1 dual-loaded LPNs are prepared and characterized as described in the Supporting Information. The anti-inflammatory effect of budesonide and the antifibrotic effect of serpine1 siRNA were evaluated at the gene level by real time polymerase chain reaction (RT-qPCR). RAW 264.7 and THP-1 macrophage cells differentiated to M0 macrophages with PMA (passage <10) were seeded in a 12-well plate (Corning, U.S.A.) at a density of 1 × 10^5^ cells per well and allowed to attach overnight. Then, cells were treated with a solution of 100 ng/mL of LPS from *Escherichia coli* (O111:B4, InvivoGen, U.S.A.) to induce inflammation for 24 h and with 10 ng/mL murine or human TGF-β1 (Abcam, U.S.A.) for 24 h to induce fibrosis. BUD (2 μg/mL), serpine1 siRNA (0.25 μg/mL), BUD + siRNA (2 and 0.25 μg/mL), empty LPNs (100 μg/mL), BUD@LPNs (equal to 2 μg/mL of BUD), siRNA@LPNs (equal to 0.25 μg/mL of siRNA), BUD@siRNA@LPNs (equal to 2 μg/mL of BUD and 0.25 μg/mL of siRNA) were added to the cells for 24 h since this is the time in which the maximum transfection efficiency is achieved and budesonide is released.^30^ Macrophages treated with LPS were used as a positive control, while cells with only cell culture medium were used as a negative control. The RNA was isolated using TRIzol reagent (Ambion, U.S.A.) and Phase Lock Gel system (5PRIME, lock Gel heavy, QuantaBio), following the manufacturer’s instructions. The cDNA was synthesized using the First-strand cDNA Synthesis Kit (Transcriptor First strand cDNA synthesis kit, Roche, Germany), and finally, the RNA was analyzed with a LightCycler 480 qPCR machine (GE Healthcare Lifescience) with Taqman chemistry. The probes used in the assay were from Thermo Fisher Scientific and predesigned: human *18s* (18s, Hs03003631_g1), murine *18s* (18s, Mm03928990_g1), murine *Nf-κb1* (Nfkb1, Mm00476361_m1), human *Nf-κb1* (Nfkb1, Hs00765730_m1), murine *Tgf-b* (Tgfb1, Mm01178820_m1), human *Tgf-b* (Tgfb1, Hs00998133_m1), murine *Tnf-a* (Tnf-α, Mm04934603_s1), human *Tnf-a* (Hs01004016_m1), murine *Serpine1* (Serpine1, Mm00435858-m1), human *Serpine1* (Serpine1, Hs00167155_m1), murine *tPa* (Plat, Mm00476931_m1), human *tPa* (Hs00263492_m1), murine matrix metalloproteinase 2 (*Mmp2*) (Mmp2, Mm00439498_m1), human *Mmp2* (Hs01548727_m1). The ΔΔCT of each sample was quantified, and the results were normalized to the housekeeping gene 18S.

### Protein Sample Preparation

RAW 264.7 and human THP-1 macrophage cells differentiated to M0 macrophages were seeded at a density of 1 × 10^6^ cells per well in a 6-well plate and were left to attach overnight. LPS (100 ng/mL) and murine or human TGF-β1 (10 ng/mL) were added for 24 h to induce inflammation and fibrosis, respectively. Afterward, without removing LPS and TGF-β1, BUD (2 μg/mL), BUD@LPNs (equal to 2 μg/mL of BUD), BUD@siRNA@LPNs (equal to 2 μg/mL of BUD and 0.25 μg/mL of siRNA, respectively) and empty LPNs (100 μg/mL) were added and left for 48 h. Cell lysis and collection of protein pellets were conducted, and the protein lysates were sonicated four times for 20 s. Protein quantification was conducted using the BCA assay (Thermo Fisher, U.S.A.). A standard curve with seven points was prepared with BSA in a concentration range between 0 and 15 μg/mL. Protein samples were prepared by mixing 30 μg of protein with 4× sample buffer + DTT at a 10:1 volume/volume ratio and adding up with lysis buffer. Samples were finally boiled at 95 °C for 10 min.

### Gel Electrophoresis and Western Blotting

A gel electrophoresis system (Bio-Rad, U.S.A.) was assembled with precast 4–20% nitrocellulose gels (Bio-Rad, U.S.A.). Thirty μg samples were loaded, and the gel was run for 45 min at 200 V. Proteins were transferred to the nitrocellulose membrane (Bio-Rad, U.S.A.) using the Turbo Transfer system (Bio-Rad, U.S.A.) followed by blocking using 5% bovine serum albumin (BSA, Sigma-Aldrich, U.S.A.) or 5% milk for 1 h at room temperature (RT) with gentle shaking. The membranes were then blocked with 5% BSA or milk and incubated with anti-NF-κβ1 antibody (Dako, U.S.A.) with a 1:1000 dilution, with anti-TGF-β1 antibody (Abcam, U.S.A., ab189778) with a 1:1000 dilution, and with anti-GAPDH antibody (Dako, U.S.A.) with a 1:10.000 dilution overnight in a cold chamber with mild shaking. Then, goat antirabbit IgG GAPDH secondary antibody (Dako, U.S.A., P0448) was incubated for 1 h at RT with mild shaking. Enhanced chemiluminescence assay (ECL) (Thermo Scientific, Pierce, U.S.A.) was used to develop the membranes, and chemiluminescence detection was performed using a BioRad machine. After each step, the membrane was washed with 1× PBS. The results were quantified using ImageJ, and the mean densitometry values were obtained and normalized to the loading control.

### Intracellular Staining for PAI-1 Quantification

RAW 264.7 and PMA-differentiated THP-1 macrophage cells were seeded in a 24-well plate at a density of 50,000 cells per well. After overnight incubation, TGF-β1 was added at 20 ng/mL to induce a fibrotic profile. Then, serpine1 siRNA-loaded LPNs and dual-loaded LPNs were added at a concentration of 100 ng/mL. Only TGF-β1 treated cells were used as the control of serpine1 (PAI-1 protein) overexpression. Empty LPNs were used as a control on cells not pretreated with TGF-β1. After 48 h incubation with the NPs, intracellular staining was performed using an Alexa Fluor 488 anti-PAI (EPR21850-82, Abcam) antibody, and the data were analyzed by flow cytometry.

### Macrophage Polarization and Cytokine Release Studies in Macrophage Cell Lines

RAW 264.7 cells were seeded in 24-well plates at a density of 50,000 cells per well. THP-1 cells were seeded in the same way but with 75 nM PMA added in the cell culture medium for 24 h to differentiate monocytes to M0 macrophages. After overnight incubation, cells were stimulated for 24 h with LPS (100 ng/mL) to induce the M1 phenotype and with IL-4 (20 ng/mL) to induce the M2 phenotype. Without removing the stimulus, BUD (2 μg/mL), BUD@LPNs (equal to 2 μg/mL of BUD), BUD@siRNA@LPNs (equal to 2 μg/mL of BUD and 0.25 μg/mL of siRNA) and empty LPNs (100 μg/mL) were added to M1 macrophages. After incubation for 48 h, the culture supernatants were collected and frozen at −20 °C for ELISA analysis, and the expression of CD86 and CD206 on the cell surfaces was detected by immunostaining with the CD80 and CD206 antibodies (BioLegend, CA, U.S.A.). The cells were washed with PBS twice and detached with a cell scrapper. The cells were centrifuged at 317*g* for 5 min and washed with PBS twice, followed by immunostaining the cell pellets with APC anti-CD86 and PE anti-CD206 at a concentration of 2 μg/mL in PBS at 4 °C for 30 min. After that, the cells were washed again with PBS twice and subsequently analyzed by flow cytometry. In each group, cells without antibody staining were used as the negative control. The fold change of MFI in each sample was calculated upon subtracting the MFI of the unstained samples and normalizing with respect to the negative control. All flow cytometry data were processed with FlowJo software. The culture supernatants were analyzed with human and murine IL-1 and human and murine IL-4 ELISA kits (PeproTech, Stockholm, Sweden) according to the manufacturer’s protocol. Triplicate samples were used for the ELISA analysis.

### Macrophage Polarization and Cytokine Release Studies in Human Primary Macrophages

Frozen CD14+ sorted monocytes from four different donors were used for differentiation to macrophages following a previously described protocol.^45^ 5 × 10^6^ monocytes per condition were differentiated to M0 macrophages by adding M-CSF (100 ng/mL) for 6 days. Then, M1 macrophages were obtained by adding LPS (2 μg/mL) and IFN-γ (200 U/mL) for 48 h, and M2 macrophages were obtained by adding IL-4 (200 U/mL) for 48 h. BUD (2 μg/mL), BUD@LPNs (equal to 2 μg/mL of BUD), BUD@siRNA@LPNs (equal to 2 μg/mL of BUD and 0.25 μg/mL of siRNA) and empty LPNs (100 μg/mL) were added to M1 macrophages for 48 h. Supernatants were collected and stored at −20 °C for Luminex cytokine analysis. Cells were detached by sucking up and down the media with a tip-bended glass pipet, and cells were divided for staining with different antibodies: CD80, CD86-PE, CD206, CD163, CD32 and VIAP primary antibodies (BioLegend, CA, U.S.A.). VIAP antibody was used as the negative control. Primary antibodies were used at a concentration of 20 μg/mL, and secondary antibody Alexa Fluor 488-labeled antigoat IgG (BioLegend, CA, U.S.A.) was used at a concentration of 20 μg/mL. Cells were resuspended in Fc-blocker (Baxter, U.S.A.) diluted 1:5 in 1× PBS, and 50 μL of the cell suspension containing 50,000 cells was used per staining tube. Twenty μL of the corresponding primary antibody was added and left incubating for 30 min at 4 °C in the dark. Two washings were done with sheath fluid, and when required, 20 μL of the secondary antibody was added and left incubating for 30 min at 4 °C in the dark. Cells were washed two times with PBS and 1% BSA and resuspended in 50 μL of this buffer for flow cytometry analysis. The pro-inflammatory cytokines IL-1β1, IL-6 and IL-12 were measured from the supernatants of the samples collected for flow cytometry using the Milliplex Human TH17 Panel and the Milliplex Human Inteferon Panel according to the manufacturer’s instructions (MilliporeSigma, U.S.A. and Canada).

### Matrix Metalloproteinase Activity Assay

RAW 264.7 and PMA-differentiated THP-1 cells were seeded in a 24-well plate at a density of 250,000 cells per well, adding PMA (75 nM) in the culture medium of THP-1 cells. After overnight incubation, media was replaced with fresh cell medium without PMA and cells were pretreated with human or murine TGF-β1 (10 ng/mL) for 24 h. Without removing the stimulus, cells were treated with serpine1 siRNA (0.25 μg/mL), siRNA@LPNs (equal to 0.25 μg/mL), BUD@siRNA@LPNs (equal to 2 μg/mL of BUD and 0.25 μg/mL of siRNA) and empty LPNs (100 μg/mL). Cells treated only with TGF-β1 were used as a positive control. After 48 h, supernatants were collected, and MMP2 activity was measured using the MMP-2 substrate (Merck). MMP-2 substrate was dissolved in DMSO/water at 1:1 v/v to prepare a 1 mg/mL stock solution. The stock was diluted to 1 mM, and 50 μL of the substrate was mixed with 50 μL of supernatants in a white, flat-bottom 96-well plate, and fluorescence was detected at 325 nm by measuring in a microplate reader.

### Activation of TLRs in Reporter Cells by Dual-Loaded LPNs

Reporter Jurkat T cells expressing individual toll-like receptors (TLRs) TLR4, TLR2/1, TLR 2/6 and TLR 2/1/6 and reporter THP-1 monocytes expressing TLR1/2, TLR2/4, TLR2/6, TLR3 and TLR7/8 were used to assess the activation of TLRs by LPNs.^52,53^ Cells were seeded in U-bottom 96-well plates at a confluence of 100.000 cells/well and were treated with BUD (2 μg/mL), BUD@LPNs (equal to 2 μg/mL of BUD) serpine1 siRNA (0.25 μg/mL), siRNA@LPNs (equal to 0.25 μg mL^–1^), BUD@siRNA@LPNs (equal to 2 μg/mL of BUD and 0.25 μg/mL of siRNA) and empty LPNs (100 μg/mL) for 24 and 48 h. PMA (100 nM) and PMA + ionomycin were used as positive controls. Then, cells were collected and washed 2 times with PBS + 1% BSA and were analyzed by flow cytometry.

### Activation of Inflammatory Factors AP.1, NF-κβ1 and NFAT in Triple Reporter T Cells by Dual-Loaded LPNs

Triple reporter Jurkat T cells transformed with plasmids that report the activation of NF-κβ1, AP.1 and NFAT factors by expressing CFP, mCherry and eGFP, respectively, were used to assess if the dual-loaded LPNs can inhibit preactivated triple reporter T cells.^54^ High-binding 96-well plates were precoated with 5 μg/mL of CD3 and CD3 + CD28 antibodies (BioLegend, CA, U.S.A.) for 24 h. The coated wells were washed, and triple reporter cells were seeded at a density of 100.000 cells/well and were put in contact with BUD (2 μg/mL), BUD@LPNs (equal to 2 μg/mL of BUD) serpine1 siRNA (0.25 μg/mL), siRNA@LPNs (equal to 0.25 μg/mL), BUD@siRNA@LPNs (equal to 2 μg/mL of BUD and 0.25 μg/mL of siRNA) and empty LPNs (100 μg/mL) for 48 h. Then, cells were collected, washed 2 times with PBS + 1% BSA, and analyzed by flow cytometer.

### Cocultures of Allogenic T Cells with LPN-Treated Macrophages

The different monocyte sets differentiated to macrophages and treated with LPNs and the corresponding controls were put in coculture with allogenic T cells. Specifically, 100.000 T cells were put in contact with 1.111, 3.333, 10.000, and 30.000 macrophage cells to do a titration. The coculture was kept for 5 days, and on day 6, H3-thymidine was added for 18–20 h. On day 7, cells were harvested using a Filtermate harvester and transferred to filter plates. Scintillation liquid solution was added, and the filter plates were left to dry for 3 h at 56 °C. The radioactive signal was read in a Beta counter (PerkinElmer 2450 microplate counter).

### Statistical Analysis

The statistical analysis was performed in GraphPad Prism 9 (GraphPad Software, Inc., La Jolla, CA, U.S.A.). A detailed description of the statistical methods used to analyze the data is reported in each figure legend. In general, ordinary one-way ANOVA followed by a Dunnett post-hoc test, ordinary two-way ANOVA followed by a Dunnett post-hoc test, and a paired Student’s *t* test were used for the statistical analyses of the different studies.

## Supporting Information Available

Preparation and characterization of empty and coloaded LPNs; gating strategy for the macrophage polarization studies; flow cytometry histograms of macrophage polarization in human primary macrophages; schematic of the reporter T cell system; pro-inflammatory cytokines analysis from supernatants of human primary macrophages; and assessment of the TLRs activation by dual-loaded LPNs at 48 h time point (PDF)
