# Supplementary material for: Study of the Synergistic Immunomodulatory and Antifibrotic Effects of Dual-Loaded Budesonide and Serpine1 siRNA Lipid–Polymer Nanoparticles Targeting Macrophage Dysregulation in Tendinopathy
Source: ACS Appl Mater Interfaces. 2024 Apr 2;16(15):18643–57. doi: 10.1021/acsami.4c02363 (PMC11040533; doi:10.1021/acsami.4c02363)
Supplement: Supplementary file 1 — am4c02363_si_001.pdf [file am4c02363_si_001.pdf]

SUPPORTING INFORMATION

Study of the synergistic immunomodulatory and anti-fibrotic effects of dual-loaded budesonide and serpine1 siRNA lipid-polymer nanoparticles targeting macrophage dysregulation in tendinopathy

Sandra López-Cerdá[1], Giuseppina Molinaro[1], Rubén Pareja Tello[1], Alexandra Correia[1], Sarojinidevi Künig[2], Peter Steinberger[2], Michael Jeltsch[1,3,4,5], Jouni Hirvonen[1], Goncalo Barreto[6,7,8], Johannes Stöckl[2]*, Hélder A. Santos[9]**

[1] Drug Research Program, Division of Pharmaceutical Chemistry and Technology, University of Helsinki, Helsinki FI-00014, Finland

[2] Centre for Pathophysiology, Infectiology and Immunology, Institute of Immunology, Medical University of Vienna, 1090 Vienna, Austria

[3] Individualized Drug Therapy Research Program, Faculty of Medicine, University of Helsinki, Helsinki FI-00014, Finland

[4] Wihuri Research Institute, Helsinki FI-00014, Finland

[5] Helsinki One Health, University of Helsinki, Helsinki FI-00014, Finland

[6] Translational Immunology Research Program, Faculty of Medicine, University of Helsinki, FI- 00014, Helsinki, Finland

[7] Orton Orthopedic Hospital, Tenholantie 10, Helsinki 00280, Finland

[8] Medical Ultrasonics Laboratory (MEDUSA), Department of Neuroscience and Biomedical Engineering, Aalto University, 02150, Espoo, Finland

[9] Department of Biomaterials and Biomedical Technology, University Medical Center Groningen, University of Groningen, Ant. Deusinglaan 1, 9713 AV Groningen, The Netherlands

*Corresponding authors: sandra.lopezcerda@helsinki.fi, johannes.stoeckl@meduniwien.ac.at; h.a.santos@umcg.nl

SUPPLEMENTARY MATERIALS AND METHODS

Preparation and characterization of empty and co-loaded LPNs.

The hybrid nanoparticles without drug (LPNs) and loaded with the drugs were prepared using an in-house manufactured glass-capillary microfluidic device following a two-steps microfluidics method, as described elsewhere [1]. Briefly, PLGA cores were formed in a first microfluidics step in which 5 mg/mL PLGA and budesonide dissolved in acetone were mixed with 1% w/v PVA. Budesonide was added in the organic phase using a 5:2.5 weight ratio of PLGA:budesonide. After purification of the PLGA cores by ultracentrifugation, the PLGA cores were resuspended in ethanol and the lipid mixture was dissolved in this organic phase. In a second microfluidics step, this organic phase was mixed at high speed with the aqueous phase of serpine1 siRNA dissolved in 1% w/v PVA, rendering budesonide and serpine1 siRNA dual-loaded LPNs. Upon purification by ultracentrifugation, the final formulation was resuspended in RNase-free milli-Q water. The size and size homogeneity (polydispersity index) of the empty and dual loaded-LPNs was controlled by dynamic light scattering (DLS) using the Zetasizer Nano ZS instrument (Malvern Panalytical Ltd., UK), as previously described.[1] The loading degree of BUD and the encapsulation efficiency of siRNA were quantified by a previously described HPLC method and Ribogreen assay, respectively.[1]

Gating strategy for the macrophage polarization studies.

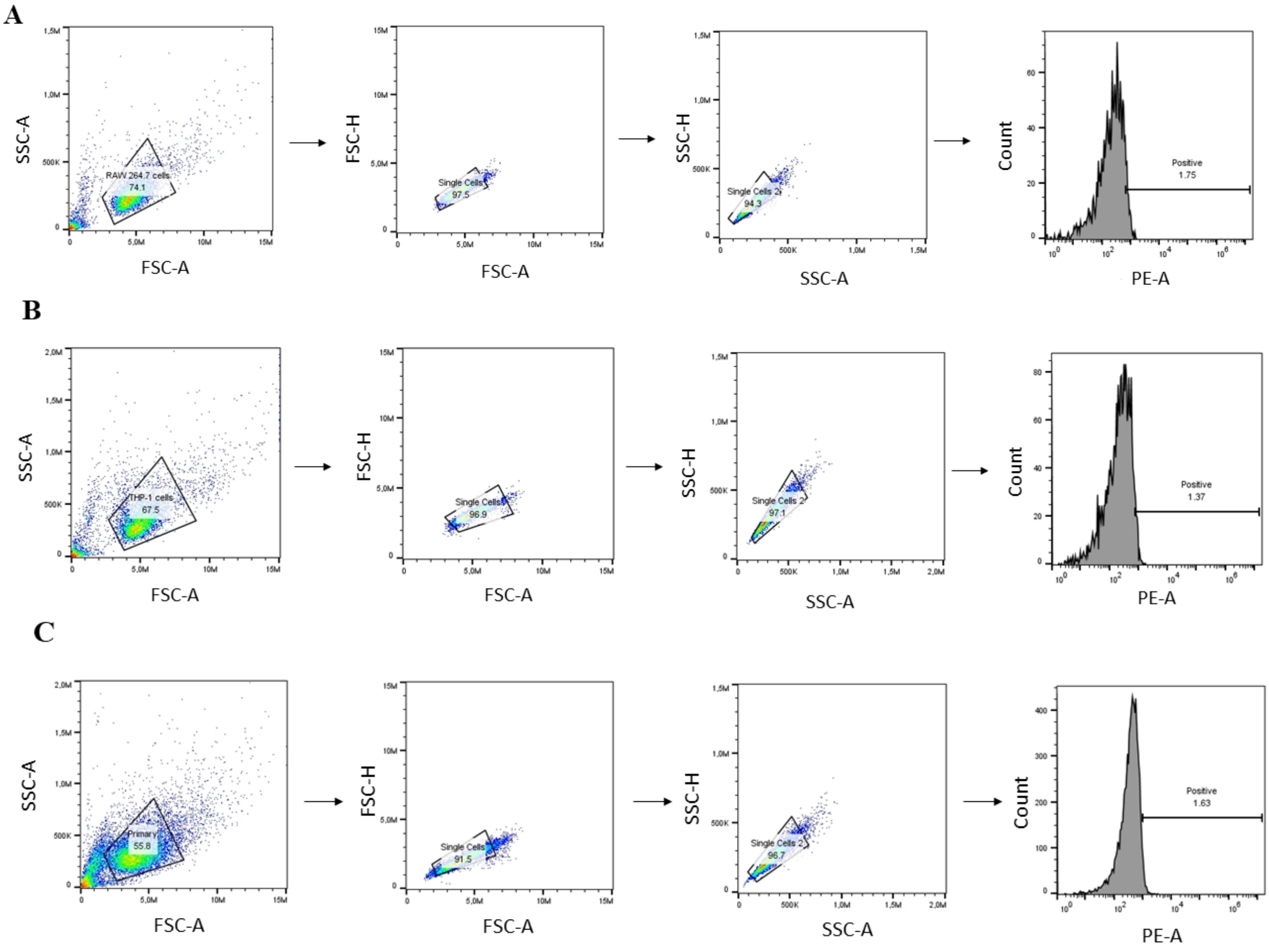

Scheme S1. Representation of the gating strategy of the negative control (cells with no antibody staining) used for the macrophage polarization studies in Figures 4 and 5. (A) Murine RAW 264.7 cells, (B) human PMA-differentiated THP-1 cells and (C) human primary macrophages, were used for the macrophage polarization studies. Primary antibodies were either directly labelled with APC or PE or a secondary antibody labelled with Alexa Fluor 488 was used, as indicated in Materials and Methods. A background signal corresponding to approximately 1.5% positive events was set as baseline for all the antibody stainings.

Schematic of the reporter T cell system

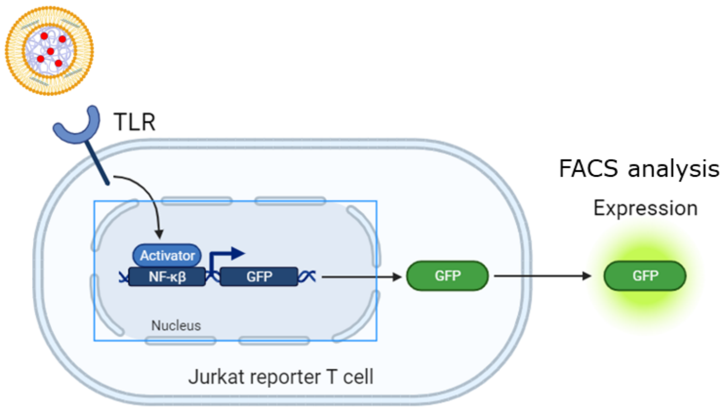

Scheme S2. Schematic representation of the reporter T cells system used to identify a potential activation of TLRs signalling by LPNs and its components using flow cytometry analysis. The reporter T cells and the reporter THP-1 monocytes were previously established elsewhere.[2,3]

SUPPLEMENTARY DATA

Flow cytometry histograms of macrophage polarization study in human primary macrophages.

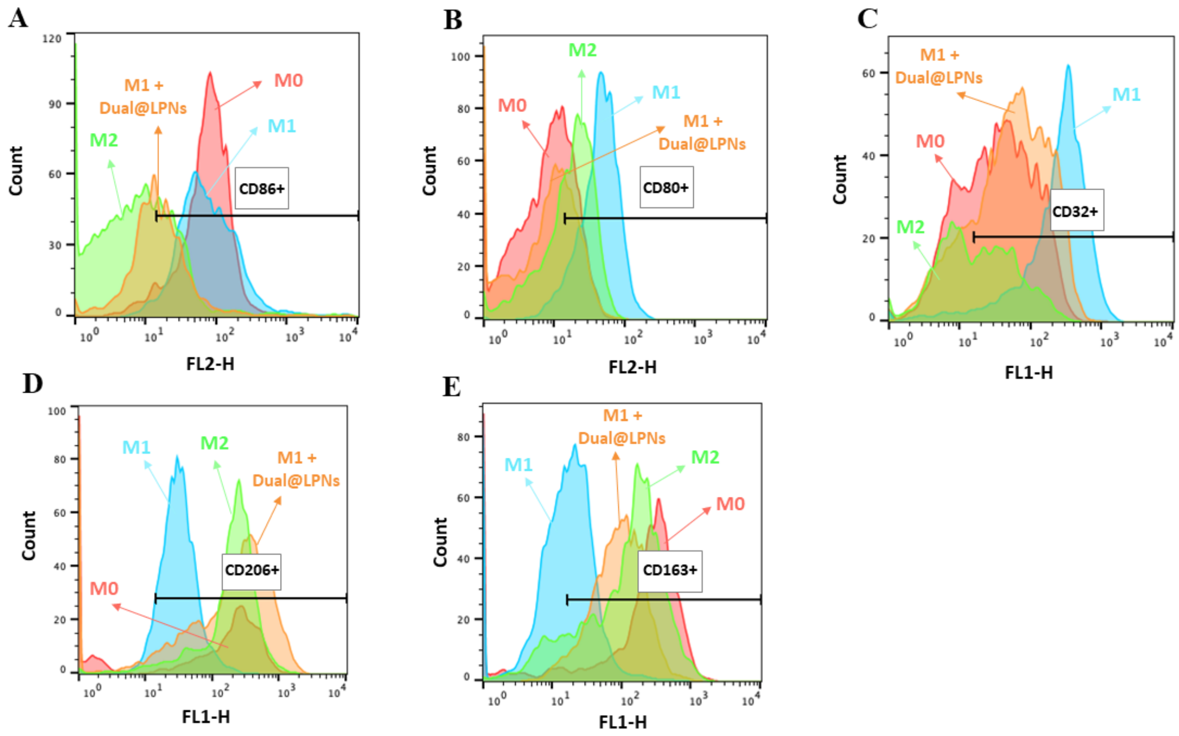

Figure S1. Overlapping flow cytometry histograms of the counts of M0, M1, M2 and M1 + dual-loaded LPNs in the macrophage polarization study with human primary macrophages. M1 macrophages are obtained by differentiation with LPS and IFN-γ and M2 are obtained by differentiation with IL-4. The graphs are comparing the expression of CD86-PE (A), CD80-PE (B), CD32 (C), CD206 (D) and CD163 (E) between those four different sample groups. For C, D and E, an Alexa Fluor 488-labelled secondary antibody was used (n≥3).

Pro-inflammatory cytokines analysis from supernatants of human primary macrophages.

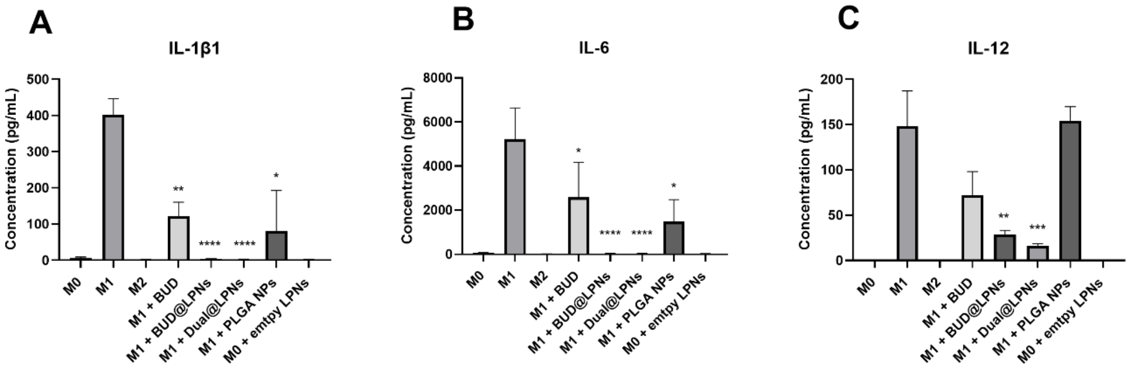

Figure S2. Analysis of the pro-inflammatory cytokines in the supernatants of the samples collected for the macrophage polarization study with human primary macrophages Data are presented as the mean ± SD (n = 4 biological replicates). A one-way ANOVA followed by a Dunnett post-hoc test was used for the statistical analysis. The significance levels of the differences were set at the probabilities of **p < 0.01 for comparing the treatment samples with the M1 positive control *p < 0.05, **p < 0.01, and ***p < 0.001.

Assessment of the TLRs activation by dual-loaded LPNs at 48 h timepoint.

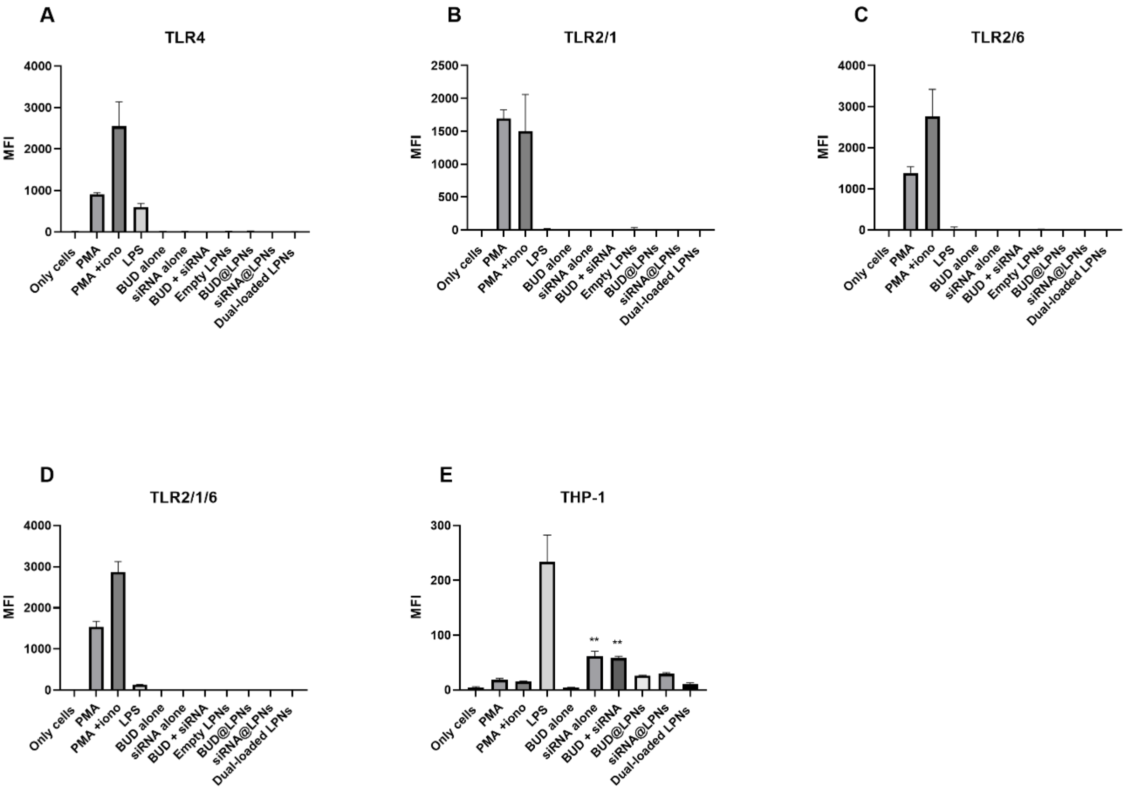

Figure S3. Activation of toll-like receptors (TLRs) in Jurkat reporter T cells by single-loaded LPNs, dual-loaded LPNs and the drugs (BUD and serpine1 siRNA alone). The cells were incubated for 48 h with the corresponding nanoparticles/drugs and cells were harvested to analyse the expression of GFP as indicator of TLR signalling activation.[2] Phorbol myristate acetate (PMA) and PMA + ionomycin were used as positive controls for (A) TLR4, (B) TLR2/1, (C) TLR2/6 and (D) TLR2/1/6 reporter T cell lines, and LPS was used as positive control for (E) reporter THP-1 monocytes. Data represents the mean fluorescence intensity (MFI) ± s.d. (n=3). A one-way ANOVA followed by a Dunnett post-hoc test was used for the statistical analysis to compare the positive controls to the treatment samples. The significance levels of the differences were set at the probabilities of *$p < 0.05$, **$p < 0.01$, and ***$p < 0.001$, to compare the negative control (only cells) with the treatment samples.

References

(1) Cerdá, S. L.; Fontana, F.; Wang, S.; Correia, A.; Molinaro, G.; Tello, R. P.; Hirvonen, J.; Celia, C.; Barreto, G.; Santos, H. A. Development of SiRNA and Budesonide Dual-Loaded Hybrid Lipid–Polymer Nanoparticles by Microfluidics Technology as a Platform for Dual Drug Delivery to Macrophages: An In Vitro Mechanistic Study. *Adv Ther.* **2023**. *6*, 8, 1-16. DOI: 10.1002/adtp.202300048.

(2) Radakovics, K.; Battin, C.; Leitner, J.; Geiselhart, S.; Paster, W.; Stöckl, J.; Hoffmann-Sommergruber, K.; Steinberger, P. A Highly Sensitive Cell-Based TLR Reporter Platform for the Specific Detection of Bacterial TLR Ligands. *Front. Immunol.* **2022**, *12,* 817604. DOI:10.3389/fimmu.2021.817604.

(3) Battin, C.; Hennig, A.; Mayrhofer, P.; Kunert, R.; Zlabinger, G. J.; Steinberger, P.; Paster, W. A Human Monocytic NF-KB Fluorescent Reporter Cell Line for Detection of Microbial Contaminants in Biological Samples. *PLoS One.* **2017**, *12* (5), e0178220. DOI: 10.1371/journal.pone.0178220.
